# Intrinsic Disorder in the BK Channel and Its Interactome

**DOI:** 10.1371/journal.pone.0094331

**Published:** 2014-04-11

**Authors:** Zhenling Peng, Yoshihisa Sakai, Lukasz Kurgan, Bernd Sokolowski, Vladimir Uversky

**Affiliations:** 1 Department of Electrical and Computer Engineering, University of Alberta, Edmonton, Alberta, Canada; 2 Department of Otolaryngology - Head and Neck Surgery, Morsani College of Medicine, University of South Florida, Tampa, Florida, United States of America; 3 Department of Molecular Medicine and Byrd Alzheimer’s Research Institute, Morsani College of Medicine, University of South Florida, Tampa, Florida, United States of America; 4 Department of Biological Sciences, Faculty of Science, King Abdulaziz University, Jeddah, Saudi Arabia; 5 Institute for Biological Instrumentation, Russian Academy of Sciences, Pushchino, Moscow Region, Russia; University of Houston, United States of America

## Abstract

The large-conductance Ca^2+^-activated K^+^ (BK) channel is broadly expressed in various mammalian cells and tissues such as neurons, skeletal and smooth muscles, exocrine cells, and sensory cells of the inner ear. Previous studies suggest that BK channels are promiscuous binders involved in a multitude of protein-protein interactions. To gain a better understanding of the potential mechanisms underlying BK interactions, we analyzed the abundance, distribution, and potential mechanisms of intrinsic disorder in 27 BK channel variants from mouse cochlea, 104 previously reported BK-associated proteins (BKAPS) from cytoplasmic and membrane/cytoskeletal regions, plus BK β- and γ-subunits. Disorder was evaluated using the MFDp algorithm, which is a consensus-based predictor that provides a strong and competitive predictive quality and PONDR, which can determine long intrinsically disordered regions (IDRs). Disorder-based binding sites or molecular recognition features (MoRFs) were found using MoRFpred and ANCHOR. BKAP functions were categorized based on Gene Ontology (GO) terms. The analyses revealed that the BK variants contain a number of IDRs. Intrinsic disorder is also common in BKAPs, of which ∼5% are completely disordered. However, intrinsic disorder is very differently distributed within BK and its partners. Approximately 65% of the disordered segments in BK channels are long (IDRs) (>50 residues), whereas >60% of the disordered segments in BKAPs are short IDRs that range in length from 4 to 30 residues. Both α and γ subunits showed various amounts of disorder as did hub proteins of the BK interactome. Our analyses suggest that intrinsic disorder is important for the function of BK and its BKAPs. Long IDRs in BK are engaged in protein-protein and protein-ligand interactions, contain multiple post-translational modification sites, and are subjected to alternative splicing. The disordered structure of BK and its BKAPs suggests one of the underlying mechanisms of their interaction.

## Introduction

The large-conductance Ca^2+^-activated K^+^ (BK) channels, also known as Slo1, MaxiK, BK_Ca_, and K_Ca_1.1 channels, are large conductance channels (100–300 pS), that act as sensors for membrane voltage and intracellular Ca^2+^, linking cell excitability, metabolism, and signaling. The *Kcnma1* gene encodes the BK α-subunit that forms homotetramers and is K^+^ selective. BK has seven transmembrane-spanning regions (S0–S6) with an extracellular N-terminus (S0) that provides a binding site for a β-subunit. Transmembrane regions S1–S4 are responsible for sensing voltage changes, while S5–S6 form a pore that conducts ions. BK has a long C-terminal region with target sequences for channel modulation, such as a Ca^2+^-bowl, composed of many positively charged amino acids, RCK1 and RCK2 domains that regulate K^+^ conductance, a tetramerization domain, LZ motifs, a heme-binding motif, phosphorylation sites, and a caveolin-targeting domain. The LZ motifs are essential for protein-protein interactions and they also modulate channel activity and expression (see [1] for recent review).

The study of structure-less proteins and protein domains/regions has taken root over the last 15 years. These studies show that there is no one specific conformation or unique structure that can describe the structural behavior of these intrinsically disordered proteins or regions (IDPs and IDPRs). Instead, IDPs/IDPRs possess highly flexible structures and exist as conformational dynamic ensembles characterized by different degree and depth of disorder [2]–[5]. Amino acid sequences of IDPs/IDPRs possess numerous specific features that make them easily recognizable from sequences of ordered proteins/regions [2], [3], [5]. These two types of sequences are so different that they are discriminated reasonably well by numerous computational tools, where comparing and combining several predictors provides additional insight regarding predicted disorder [6]–[8]. Computational analysis reveald that IDPs/IDPRs are highly abundant in almost all proteomes analyzed so far [3], [5], [9]–[11]. They have a number of crucial biological functions related to regulation, signaling, and control pathways, representing an important complement to the functional diversity of ordered proteins (see [5], [12], [13] for reviews). Due to their conformational plasticity and flexibility, and interactions with multiple unrelated partners, IDPs and IDPRs are often seen as major mediators, regulators, and controllers of various protein interaction networks (see [5], [12], [13] for reviews). An intriguing feature of IDPs/IDPRs is their ability to adopt different conformations when interacting with different binding partners [14]. They can serve as chameleon sequences that fold differently depending on the environment or the binding partner. Intrinsic disorder is abundantly found in proteins associated with pathogenesis of various human diseases, including cancer, diabetes, and neurodegenerative and cardiovascular diseases, among others (see [15] for review).

Intrinsic disorder is widely common in integral membrane proteins [16]–[20]. For example, in human integral plasma membrane proteins, predicted disorder was estimated three times more frequently on the cytoplasmic surface compared to the external surface of these proteins. Furthermore 40% of these proteins were predicted to contain long IDPRs, whereas only 5% of *E. coli* membrane proteins were predicted to have such regions [18]. A prediction study on single-pass type I transmembrane proteins was in agreement with the aforementioned study [18], since the cytoplasmic domains of these proteins were highly enriched in charged residues (R, E, K), accounting for the disorder in these regions [19]. Also, a systematic computational analysis of structurally characterized integral membrane proteins revealed that current algorithms make reasonably accurate predictions of disorder for these proteins. This accuracy occurs despite the fact that disordered regions from helical bundle and β-barrel integral membrane proteins, and those from water soluble proteins, all exhibit statistically distinct compositional biases in amino acids [21].

BK channels are important for sensory or hair cell “tuning” in the hearing endorgan of lower vertebrates, whereas in mammals this channel increases hearing sensitivity in the cochlea. Recent studies show that the BK channel interacts with a number of different proteins in the cochlea [22], [23]. Moreover, it was found that there are many variants of this channel in the developing and adult mouse cochlea [24]. To better understand the mechanisms that underlie BK-BKAP interactions, we examined their intrinsic disorder and include those of the α- and γ-subunits. Here, we use a set of bioinformatics tools to discern the IDPRs of these proteins and their related functions.

## Materials and Methods

### Datasets

No animals were used in this study, only previously published sequences of 27 BK channel variants for mouse cochlea [24], together with 104 and 71 published proteins [22], [23] that interact with the BK channel in the membrane/cytoskeletal and cytoplasmic regions, respectively. We successfully mapped 22 BK channel variants, 97 membrane/cytoskeletal and 64 cytoplasmic partners of BK channel into UniProKB [25] to facilitate bioinformatics analysis. The 22 variants are categorized into three types, depending on the last three amino acids in their C-terminus: DEC type with 6 chains, VYR with 9 chains, and ERL with 7 sequences. In addition, we analyzed β-subunits KCNMB1 (Q8CAE3), KCNMB2 (Q9CZM9), KCNMB3 (E9Q7U0), and KCNMB4 (Q9JIN6), and γ-subunits LRRC26 (Q91W20), LRRC38 (A2VDH3), LRRC52 (Q5M8M9), and LRRC55 (Q3UY51).

### Characterization of Eukaryotic Linear Motifs (ELMs)

We extracted 59 classes of eukaryotic linear motifs (ELM) for the 22 BK channel variants using the ELM database [26] from July 2013. The ELM resource has been available for over 10 years and provides access to high-quality manually curated annotations [27]. We grouped these 59 classes into 4 ELM types including cleavage sites (CLV), ligand binding sites (LIG), sites of posttranslational modification (MOD) and subcellular targeting sites (TRG) [26]. The ELM annotations are available in Table S1.

### Intrinsic Disorder

Disorder was predicted using the MFDp method [28], which is a consensus-based predictor that was recently shown to provide strong and competitive predictive quality [29]–[31]. For instance, in a recent benchmark experiment [31], MFDp was shown to provide predictions with area under the ROC curve (AUC) and Matthews correlation coefficient (MCC) values of 0.89 and 0.49, respectively. We calculated the disorder content (i.e., fraction of disordered residues in a given protein chain) and the number of disordered segments; we assume that a disordered segment includes at least 4 consecutive disordered residues, which is consistent with [32]–[34]. We also counted the number of long disordered segments that consists of at least 30 consecutive disordered residues; such long segments are implicated in protein-protein recognition [35].

### MoRF Regions and AIBSs

MoRFpred, which is the leading predictor of molecular recognition features (MoRF) [36], was used to annotate MoRF regions. This method generates relatively accurate predictions, which were estimated to achieve an accuracy of 94% and AUC of 0.67 [36]. MoRFs are short (5 to 25 amino acids) disordered regions that undergo disorder-to-order transition upon binding to protein partners that are implicated in signaling and regulatory functions [5], [37]–[39]. Following Mohan *et al*. [37], we categorized MoRF regions into α-MoRFs (fold into α-helices), β-MoRFs (fold into β-strands), γ-MoRFs (coils) and complex-MoRFs (mixture of different secondary structure) based on the secondary structure predicted with the PSI-PRED method [40].

In addition to the MoRF predictor, which finds disorder-driven binding sites as described above, the ANCHOR algorithm was used to identify potential binding sites in disordered regions [41], [42]. This method was shown to provide predictions with accuracy of about 70% [42]. Methodologically and logistically, ANCHOR is very different from the MoRFPred. It relies on a pairwise energy estimation approach developed for the general disorder prediction method IUPred [43], [44]. ANCHOR is based on the hypothesis that long regions of disorder contain localized potential binding sites that cannot form enough favorable intrachain interactions to fold on their own, but are likely to gain stabilizing energy by interacting with a globular protein partner [41], [42]. Therefore, ANCHOR predicts disordered binding regions by identifying segments, referred to as ANCHOR-identified binding sites (AIBSs) that reside in disordered regions, based on these two hypothetical attributes.

### Characterization of BK Channels

Since the considered 22 BK channels variants are very similar in their sequences and differ primarily through alternative splicing events in the *kcnma1* gene, we aligned them based on multiple sequence alignment (MSA) with ClustalW [45] using default parameters (i.e., gap_open = 10, gap_extend = 0.1, and Gonnet protein weight matrix). For these 22 BK channel variants, we provide majority vote-based profiles, which are based on the consideration of a given annotation if it occurs in at least 50% of variants at a given position in the chain. These profiles were used to analyze disordered and MoRF regions, globular domains and the four types of ELMs. Globular domains were annotated using Pfam [46] and SMART [47]. Profiles are given in Table S2, while raw data (per chain) are found in Table S3.

### Evolutionary Conservation

The evolutionary conservation of the sequence was quantified with relative entropy [48] that was calculated from the Weighted Observed Percentages (WOP) profiles generated by PSI-BLAST [49] for each BK channel variant. PSI-BLAST was run with default parameters (-j 3, -h 0.001) against the nr database. Higher values of relative entropy indicate a higher degree of conservation. The use of relative entropy is motivated by work in [48] that suggests that it leads to more biologically relevant results compared to some other conservation scores. Moreover, it was recently applied to investigate disorder in histones [50], ribosomal proteins [51], and cell death cycle proteins [52], and to identify nucleotide-binding residues [53] and catalytic sites [54]. We summarize the conservation across the 22 variants by averaging their conservation score for each aligned position. Raw data are available in Table S4.

### Functional Annotation of Disordered Regions

Following the protocol from [51], [52], we predicted functions of the disordered segments. The protocol is based on a local pairwise alignment against functionally annotated disordered segments collected from DisProt version 6.02 [55]. We aligned each of the 441 disordered segments into the set of 862 disordered segments collected from the DisProt database that have functional annotations. These include 94, 125 and 222 disordered segments extracted from the 22 BK channel variants, and the 64 cytoplasmic and 97 membrane/cytoskeletal partners, respectively. We calculated alignment using the Smith-Waterman algorithm [56] using the EMBOSS implementation with default parameters (gap_open = 10, gap_extend = 0.5, and blosum62 matrix). We defined sequence similarity as the number of identical residues in the local alignment divided by the length of the local alignment or by the length of the shorter of the two aligned segments, whichever is larger. We transferred the annotation if the similarity was greater than 80%, which should assure the generation of only high quality predictions. Consequently, we successfully annotated 89 disordered segments with the 19 functions that are listed in Table 1. These functions included 26, 27, and 36 from BK, cytoplasmic, and membrane/cytoskeletal partners, respectively.

**Table 1:** A list of functional annotations with corresponding number of disordered segments.

Function	Number of Disordered Segments		
	Membrane/Cytoskeletal Partners	Cytoplasmic Partners	BK Variants
Protein-protein binding	25	20	7
Substrate/ligand binding	5	5	26
Protein-DNA binding	8	6	7
Flexible linkers/spacers	7	4	3
Phosphorylation	2	8	3
Intra-protein interaction	3	2	3
Protein-lipid interaction	6	2	0
Metal binding	4	3	0
*Nuclear localization*	*1*	*3*	*0*
*Polymerization*	*1*	*2*	*0*
*Electron transfer*	*2*	*1*	*0*
*Entropic spring*	*2*	*1*	*0*
*Transactivation*	*2*	*1*	*0*
*Apoptosis Regulation*	*0*	*1*	*0*
*Cofactor/heme binding*	*0*	*1*	*0*
*Glycosylation*	*0*	*1*	*0*
*Protein-tRNA binding*	*0*	*1*	*0*
*Autoregulatory*	*1*	*0*	*0*
*Entropic bristle*	*1*	*0*	*0*

The list shows 27, 36 and 26 disordered segments from membrane/cytoskeletal and cytoplasmic partners, and from BK channel variants, respectively. The functions are sorted in descending order by total number of segments. Functions shown in italics were annotated for less than 5 segments.

### Analysis of Disorder Propensities

Disorder propensities were evaluated using the members of the PONDR family of intrinsic disorder predictors. Here, scores above 0.5 correspond to disordered residues/regions. PONDR® VSL2B [57] is one of the most accurate stand-alone disorder predictors, with recently reported AUC and MCC values of 0.79 and 0.40, respectively [30]. PONDR® VL3 possesses high accuracy in finding long IDPRs [58]. PONDR® VLXT is not the most accurate predictor but has high sensitivity to local sequence peculiarities, which are often associated with disorder-based interaction sites [59]. In contrast, PONDR-FIT represents a metapredictor that is one of the most accurate disorder predictors, since it is moderately more accurate than each of the component predictors [60].

### Statistical Analysis

Statistical significance of differences was determined with a t-test for data with a normal distribution; otherwise, we used the Wilcoxon rank sum test. Data normality was evaluated with the Anderson-Darling test set at a significance level of 0.05. For measurements computed per proteins, such as disorder content, number of disordered and long disordered segments, number of MoRF regions, we assessed significance of differences between two populations of these per protein values (e.g., BK channels and their cytoplasmic partners). For measurements computed over a population of proteins, (e.g., number of fully disordered proteins and number of proteins with a given putative function), we determined significant differences between two populations of these measurements computed over ten subsets with 50% of the proteins in each population selected at random. We also report median and 25^th^ and 75^th^ centiles of these measurements since a majority of the data was not normally distributed.

## Results

### Characterization of Intrinsic Disorder

**Figure 1:**
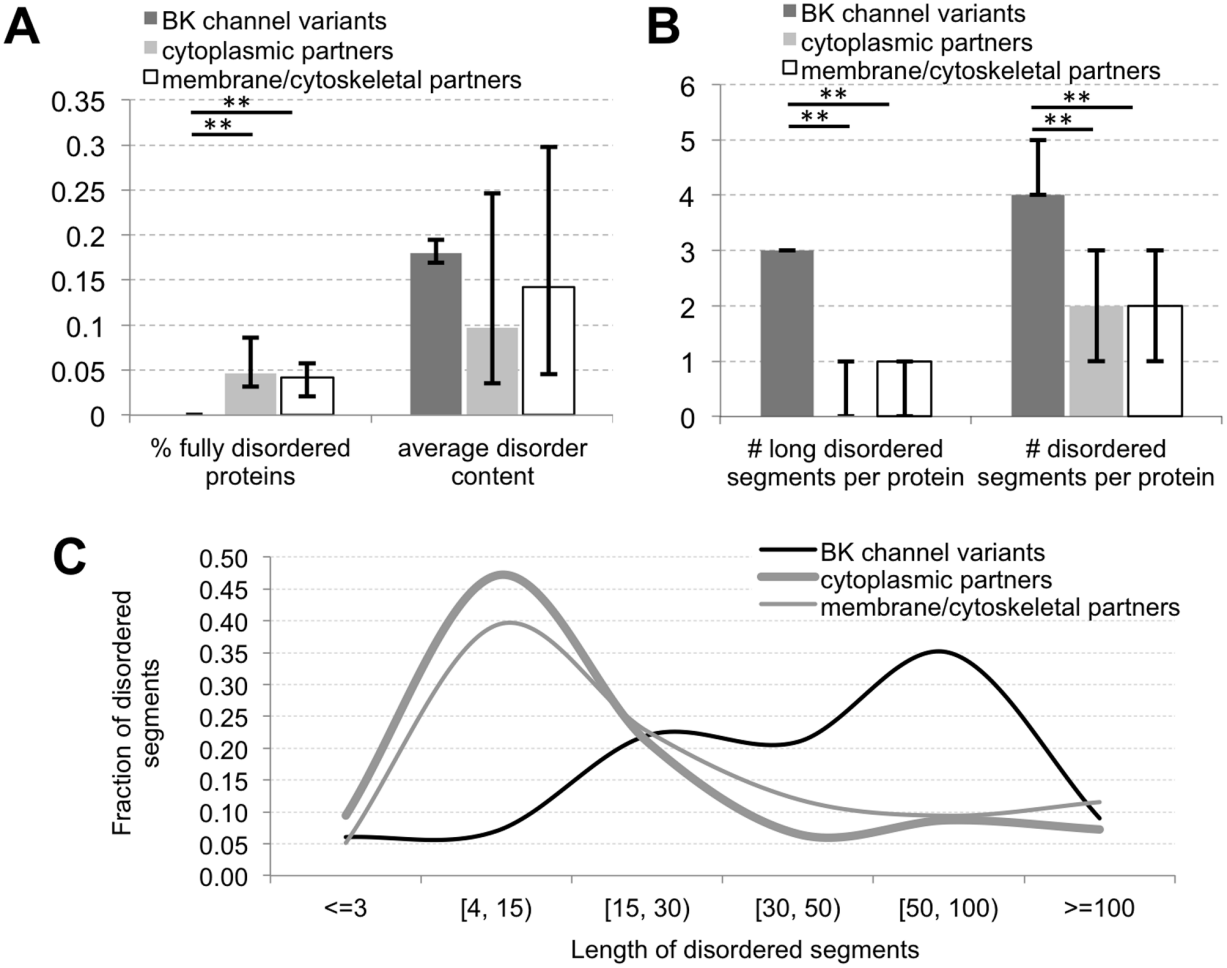
Overall characterization of intrinsic disorder in BK and BKAPs. (**A**) Fraction of predicted fully disordered proteins and average disorder content. (**B**) Number of predicted disordered segments and long (30 or more consecutive residues) disordered segments per protein. (**C**) Distribution of the length of putative disordered segments. The bar charts in panels A and B report median values with the corresponding 25^th^ and 75^th^ centiles (shown as error bars). *p<0.01, **for *p*-value<0.001.

The median values for putative disorder content (i.e., the fraction of disordered residues) of the BK channel variants and their BKAPs is 18, 10 and 14%, respectively (Figure 1A). These values are not significantly different since some BKAPs have a high disorder content. The content values of the BK channels are typical for eukaryotic organisms, since the estimated average disorder content of the mouse proteome is 22% [61]. While the BK channel chain is largely structured, about 4% of its BKAPs are fully disordered, i.e., the entire protein is composed of disordered residues (Figure 1A). The median number of putative disordered regions in BK channel variants is four per protein, where three of them contain 30 or more residues (Figure 1B). To compare, the median number of disordered regions in BKAPs is two per chain, and one or less long regions per chain. The difference in the number of the disordered regions is statistically significant (Figure 1B). A more detailed analysis of the distribution of length of the putative disordered segments (Figure 1C) shows that about 65% of the disordered segments in BK variants are long (30 or more consecutive residues). In comparison, 62% of the disordered segments in BKAPs are short (up to 30 consecutive amino acids). Statistical analyses of length differences in disordered regions reveals that BK channels have longer regions compared to both cytoplasmic and membrane/cytoskeletal BKAPs (*p*<0.001). However, as the distributions in Figure 1C suggest, the length of the disordered segments in the two partner types is relatively similar (*p* = 0.014). The BK channel chains are relatively long with over 1100 residues, which is about three times longer than the sequences of BKAPs. To summarize, our analysis indicates that proteins that interact with BK have a lower number of putative disordered regions compared to BK, and that most of these regions are shorter.

### Characterization of MoRF Regions

**Figure 2:**
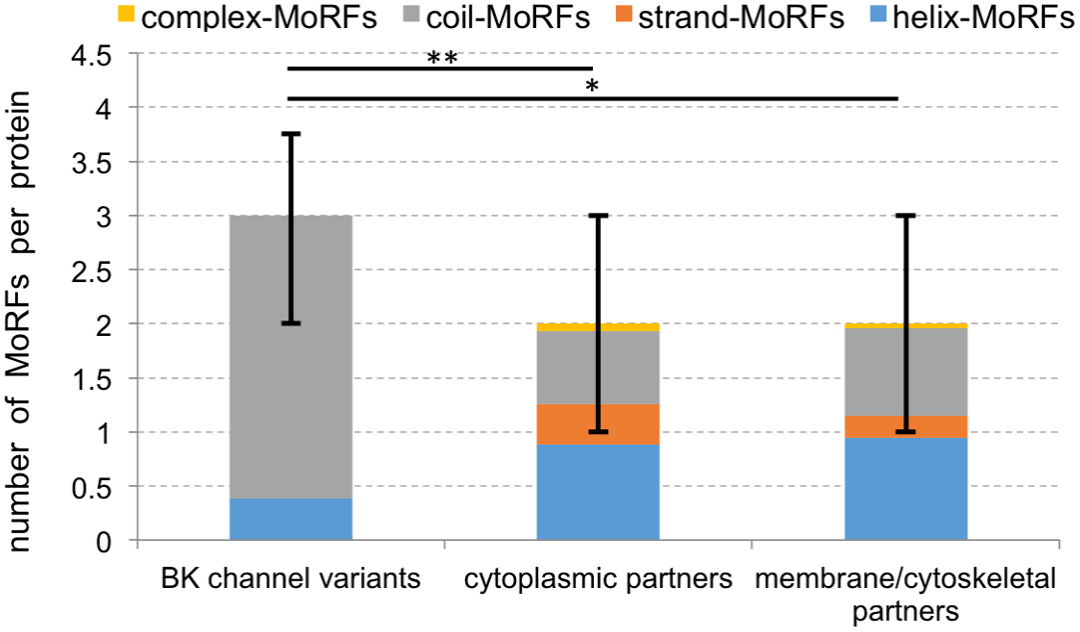
Number of predicted MoRF regions per protein for BK channel variants and cytoplasmic and membrane/cytoskeletal BKAPs. The bars are subdivided into different colors to denote MoRF types. The bar chart shows median values with the corresponding 25^th^ and 75^th^ centiles (shown as error bars). *p<0.01; **for *p*<0.001.

Analysis of BKAPs shows that the majority of their disordered segments are composed of 4 to 30 consecutive disordered residues (Figure 1C). This finding underlies our analysis of MoRF regions [5], [37]–[39], which are defined as short (5 to 25 residues) disordered regions that undergo disorder-to-order transition upon binding to protein partners. The median number of three putative MoRF regions per protein in BK variants is higher (*p*<0.005) than the median of two regions found in cytoplasmic and cytoskeletal BKAPs (Figure 2). Moreover, differences were found when classifying these putative regions into the four MoRF types, as defined by conformational changes assumed upon binding. These types include helix (α-MoRFs), strand (β-MoRFs), coil (γ-MoRFs) and complex-MoRFs, which combine multiple secondary structure types. BK has a larger fraction of γ-MoRFs, whereas BK partners have more α- and β-MoRFs. More specifically, ∼13% and 87% of MoRFs in BK fold into coils and helices, respectively. In contrast, 34% and 44% of cytoplasmic BKAPs and 41% and 47% of membrane/cytoskeletal BKAPS fold into these respective structures. The enrichment in putative γ-MoRFs in BK is statistically significant (*p*<0.001) when compared to BKAPs. However, membrane/cytoskeletal BKAPs have more putative α-MoRFs than BK variants (*p*<0.01) and cytoplasmic and membrane/cytoskeletal BKAPs have β-MoRFs that are not present in BK (*p*<0.01 and *p*<0.025, respectively). This result suggests that BK partners are possibly involved in different types of disorder mediated protein-protein interactions compared to the BK channel.

### Overall Characterization of BK Channel

**Figure 3:**
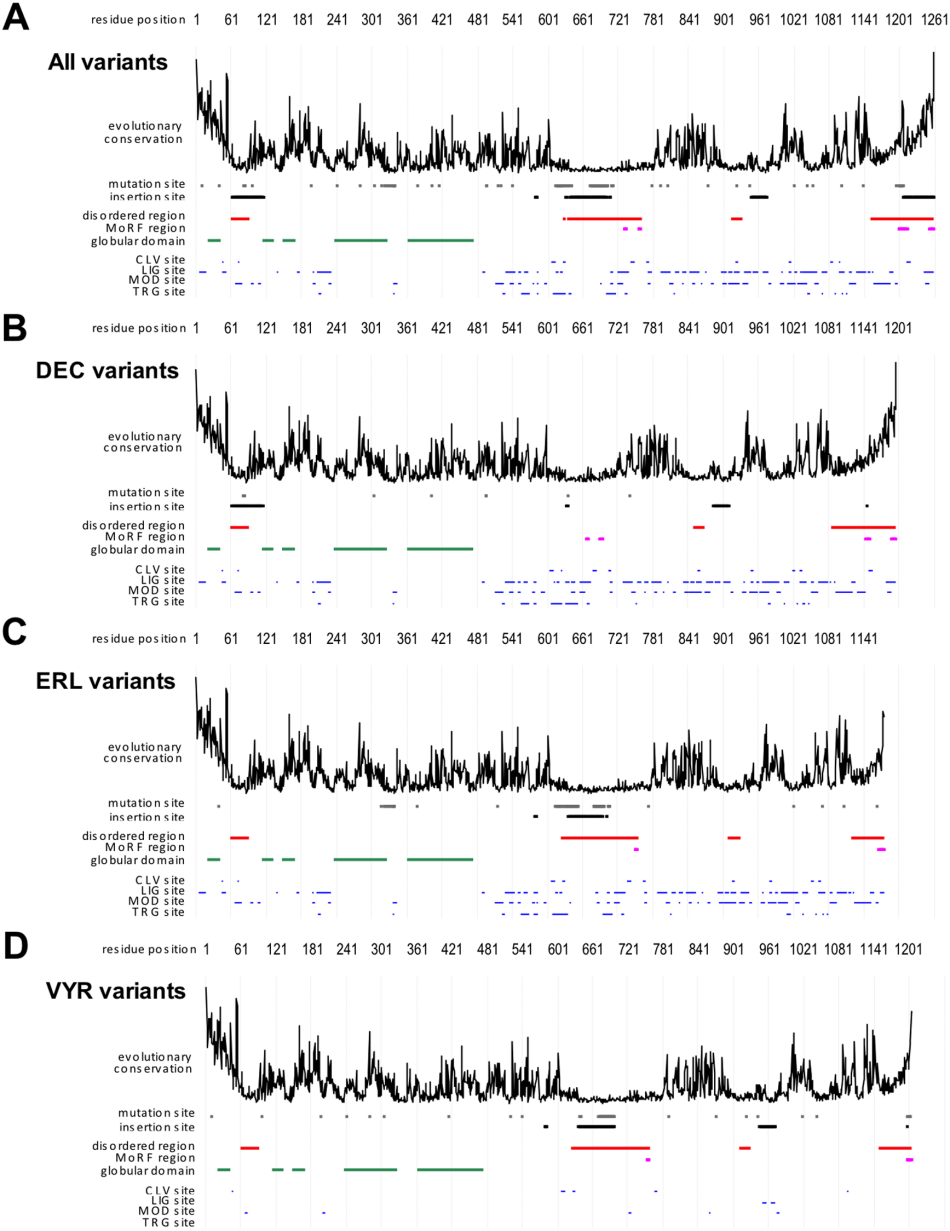
Overall characterization of BK channel variants. Sequence profiles of BK for (**A**) all 22 considered variants, (**B**) 6 DEC type variants, (**C**) 7 ERL type variants, and (**D**) 9 VYR type variants. Profile includes sequence conservation (*uppermost plot*), sites of mutations between different variants (*gray*), regions inserted/deleted through alternative splicing (*black*), majority-vote based (a given annotation is considered if it occurs in at least 50% of variants at a given position in the chain) profiles for putative disorder (*red*), MoRFs (*pink*), globular domains (*green*), and four types of ELMs (*blue*). ELMs include cleavage sites (CLV), ligand binding sites (LIG), sites of posttranslational modification (MOD) and subcellular targeting sites (TRG).

The multiple sequence alignment (MSA) of the BK variants reveals that they are similar in their sequence, but differ primarily due to alternative splicing events. Specifically, 78.3% of the residues are identical between the 22 variants, 16.4% positions differ as a consequence of an insertion through alternative splicing, and 8.1% differ at individual positions due to point mutations (Figure 3A). We note that mutation and insertion sites overlap and thus total>100%.

We also annotated putative disordered and MoRF regions, globular domains, and four types of ELMs in the BK variants (Figure 3) using majority vote. Figure 3 also includes evolutionary conservation, which is based on averages across the considered BK variants. The raw data (per chain) are provided in Supplementary files 3 and 4. We observe that MoRF regions are located within disordered regions, which is expected, as MoRFs are short functional disordered regions. The ELMs do not overlap with the annotated globular domains and this is consistent with the fact that ELMs are annotated outside of the globular domains [26].

The disordered and MoRF regions as well as the insertion sites are located outside of the globular domains. Interestingly, the insertion segments are co-localized with the disordered segments. The three largest insertion segments are predicted as disordered and 73% of the residues at the insertion sites are disordered (black and red horizontal lines, Figure 3A). This demonstrates that alternative splicing events are associated with inclusion of disordered regions in the BK channel. A particularly interesting example is the C-terminus tail, which is predicted as disordered in all 22 variants, including the elongated (through splicing) terminus of the BK DEC type (Figure 3B). This tail includes two conserved MoRF regions that facilitate interactions with other proteins or peptides, where one MoRF is exclusive to the DEC type (Figures 3B, C, and D).

The different BK C-terminus types share similar annotations with globular domains, disordered and MoRF regions, ELMs, and evolutionary profiles (Figures 3B, C, and D). Notable differences include: ERL and VYR types have a shorter disordered conserved tail that lacks one MoRF region compared to the DEC type; the DEC type lacks a long disordered region in the middle of the chain, due to the exclusion of a corresponding insertion site, through alternative splicing, found in the ERL and VYR sites; the VYR type has a depletion in ELM annotations.

The BK channel includes 42 ligand-binding sites (LIG), 34 posttranslational modification sites (MOD), 16 subcellular targeting sites (TRG) and 12 cleavage sites (CLV) based on ELMs (blue horizontal lines Figure 3A). On average, close to 30% of these ELMs are disordered (Table 2), which is consistent with the fact that functional ELMs occur preferentially in disordered regions [62]. Moreover, a substantial fraction of ELMs, between 25% and 38% depending on the ELM type (Table 2), occur in mutation sites. To compare, about 11% of all mutations occur in globular domains compared with 45% that are located in ELM regions. This outcome suggests that the functions associated with these ELMs are modulated between different BK channel variants. On the other hand, mutation rates are much lower in the four MoRF regions. Three regions are identical between channel variants that include them and only one region near the C terminus that includes one mutation site.

**Table 2:** Characterization of rates of mutation and disorder, and average evolutionary conservation for different ELMs found in BK.

ELM Type	Mutations (%)	Disordered (%)	Average conservation
CLV	25.0	41.7	0.72
LIG	28.6	23.8	0.98
MOD	26.5	26.5	0.81
TRG	37.5	25.0	0.80
All types	31.9	29.8	0.97

ELMs are subdivided into cleavage sites (CLV), ligand binding sites (LIG), posttranslational modification sites (MOD), and subcellular targeting sites (TRG).

The overall evolutionary conservation of the BK channel is moderate, since the average value over all residues equals 0.95. As expected, the mutation sites have a low average conservation at 0.65, with the conservation of the mutation sites located in the ELM regions at 0.58. MoRF regions are highly conserved, with an average score of 1.68. Residues in the globular domains are more highly conserved (1.07) than the disordered residues (0.83) and the ELM sites (0.97). A breakdown of conservation scores across the four ELM types is in Table 2. We also observe that disordered residues in ELM sites are more highly conserved (0.90) compared to the remaining disordered amino acids (0.75). Interestingly, both termini of the BK channel are highly conserved, including the disordered C-terminus that has two MoRF segments.

### Functional Analysis of Disordered Segments

**Figure 4:**
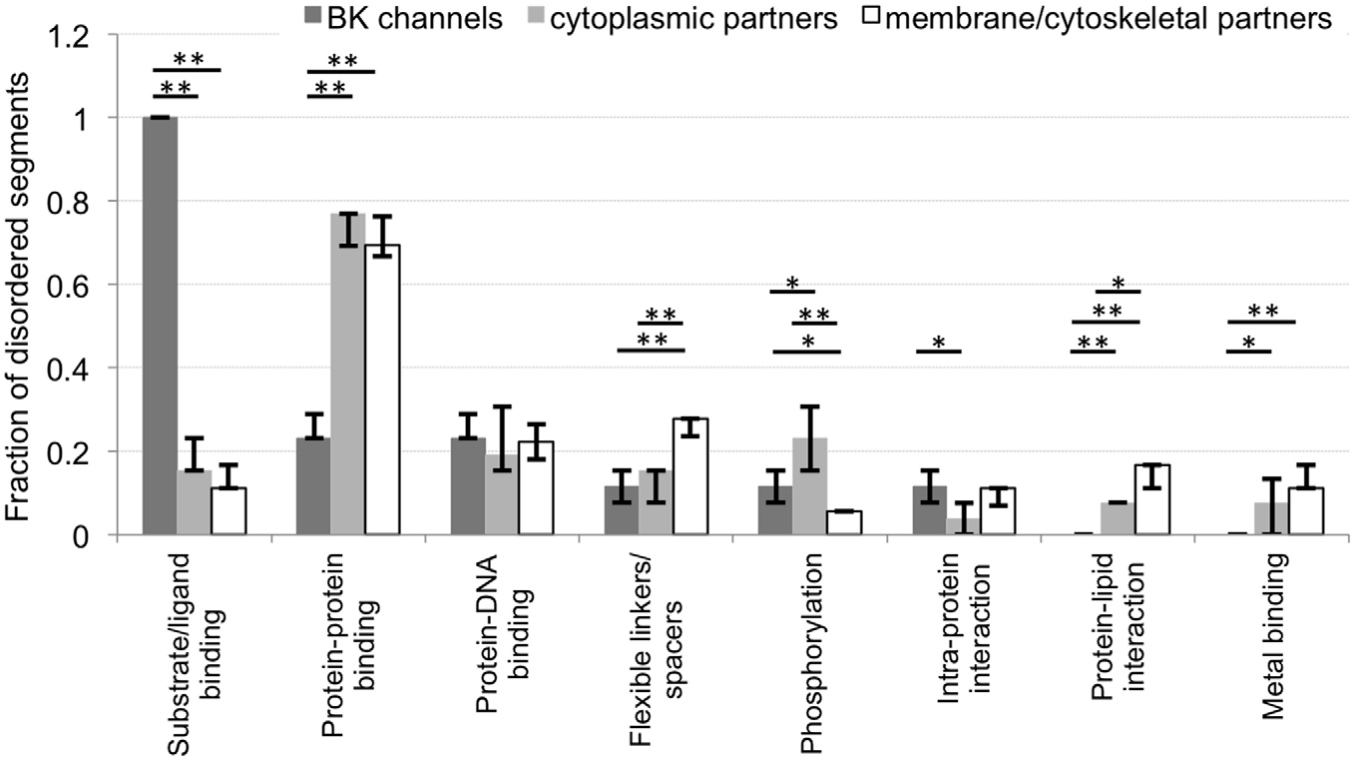
Characterization of putative functions for disordered regions using Gene Ontology (GO). Fraction of disordered segments for a given function is grouped by three protein sets, including BK and cytoplasmic and membrane/cytoskeletal BKAPs. The eight functions are sorted by decreasing number of disordered segments from the BK channel. The bar chart reports median fraction of disordered regions with a given putative function with the corresponding 25^th^ and 75^th^ centiles (shown as error bars). *p<0.01, **p<0.001.

BK channels and their partners include a substantial number of disordered regions and their sizes differ substantially between the channel and the partners (Figure 1). Using annotation protocols from [51], [52], we enumerate and contrast putative functions of these disordered segments. We found 19 functions (Table 1) and our analysis focuses on the eight functions that are annotated for at least 5 segments. Figure 4 compares putative functional annotations of disordered segments between BK and its cytoplasmic and membrane/cytoskeletal partners.

The results suggest that disorder plays important and diverse functional roles, from facilitation of the various binding events including protein-protein, -DNA, -lipid, -metal and intra-protein interactions, to implementation of flexible linkers and involvement in phosphorylation. The disordered segments from both types of BKAPs share similar functions, since they are involved primarily in interactions with proteins, DNA, metals and intra-protein interactions. In a few instances the abundance of their putative functional annotations differs. We note a higher rate of putative disordered regions in phosphorylation sites of the cytoplasmic partners, and enrichment of protein-lipid interaction annotations and linker/spacer regions in the membrane/cytoskeletal partners. The disordered segments found in the BK channel are predicted to be involved in six out of the eight functions, thus excluding protein-lipid interactions and metal binding. The most common predicted function of these regions is in substrate/ligand binding rather than in protein-protein binding, which is the dominant function of the disordered segments in BKAPs.

### Disorder in Known BK Structures, Isoforms, and BKAPs

**Figure 5:**
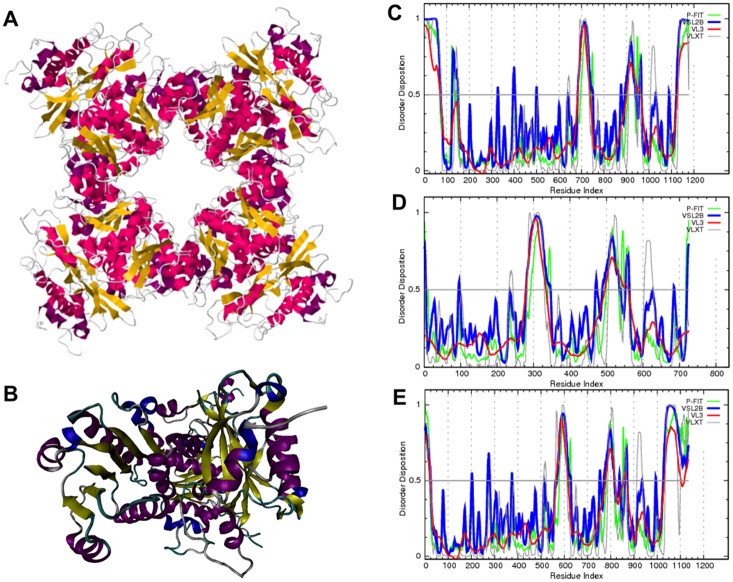
Comparisons of BK intrinsic disorder with BK crystal structures. (**A**) Crystal structure of the intracellular C-terminal domain of human BK in a form showing the gating ring constituted as a modified tetramer (PDB ID: 3NAF) [68]. (**B**) A crystal structure of the non-modified monomer of this domain (PDB ID: 3MT5) [63]. Comparison of disorder profiles determined for: (**C**) the construct used in the crystallization experiments of residues 341–1056 [63] (numbered in plot as 1–715), (**D**) isoform-5 of human BK (UniProt ID: Q17921-5), and (**E**) the DEC variant of mouse BK (UniProt ID: C3VLD3). Disorder propensities were predicted using four members of the PONDR family depicted as different colors.

Crystal structures for the tetrameric complex of the human BK intracellular subunits known as the intracellular gating ring (PDB ID: 3NAF, [63]) and the BK intracellular domain (aa 341–1065), showing the monomeric form of this domain (PDB ID: 3MT5, [57]), are illustrated in Figures 5A and B, respectively. The first was obtained using a construct containing a human BK intracellular domain starting from Arg 3 to the C-terminus with deleted residues 633–669. This form included an extra 32-amino-acid-long sequence from a mutant transcription factor GCN4 leucine zipper (GCN4_LI), known to form a four-helical coiled-coil domain, inserted before the N-terminus of the BK intracellular domain [63]. The second crystallization used a construct of the human BK intracellular domain with residues 340 to 1065, to provide a structural description of the monomeric form of this domain [63]. (Figure 5B). The crystal structure defines two tandem regulator of conductance for K^+^ (RCK) domains in the C terminus, with each domain having a bi-lobed shape. Here, the larger N-terminal lobe of each RCK domain forms a Rossmann fold attached to a smaller C-terminal lobe via a helix-turn-helix connector [63]. The very terminal end residues (∼55) of the human protein were not used in these crystallization experiments.

**Figure 6:**
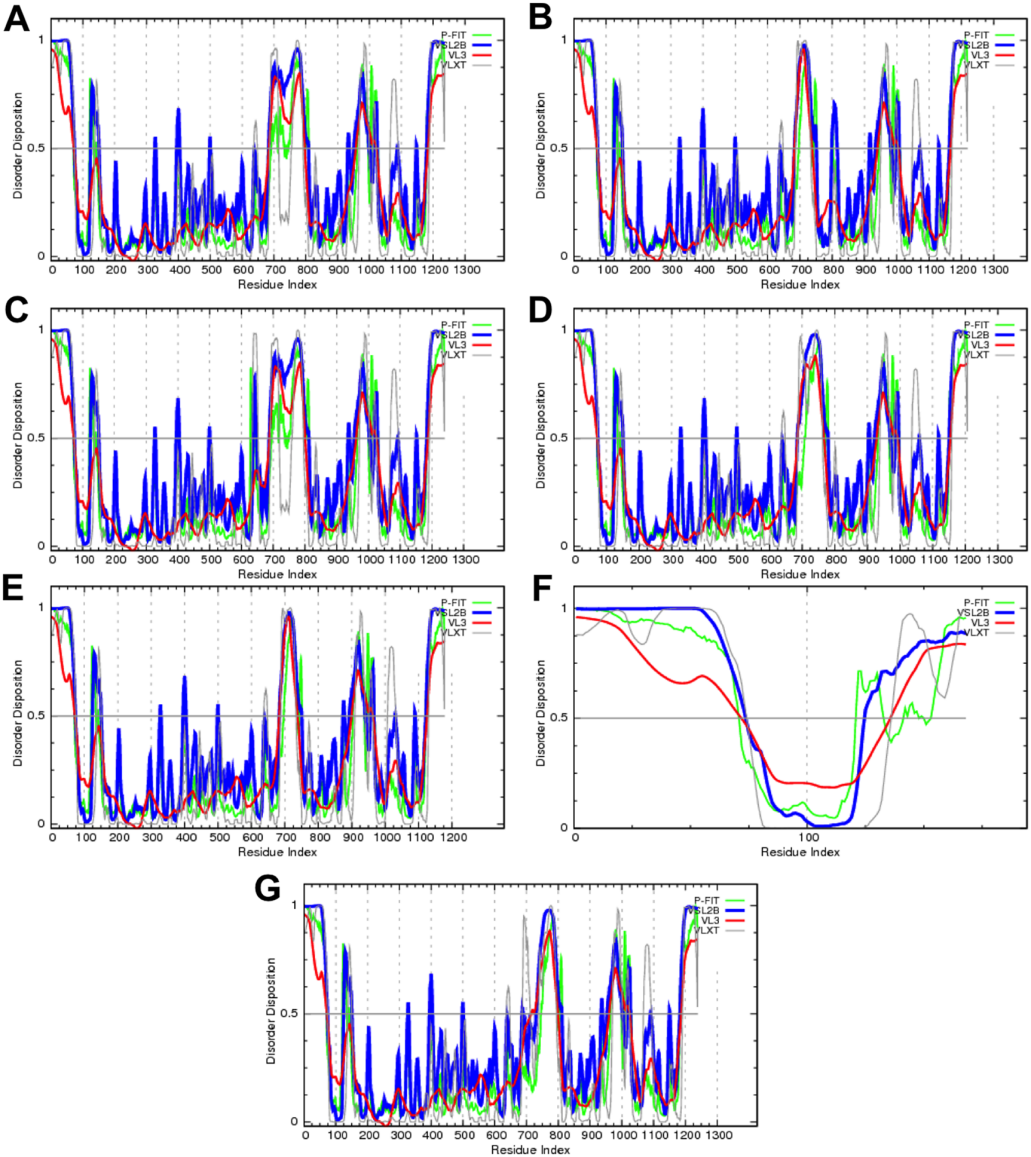
Effect of alternative splicing on the disorder propensity of human BK. Disorder profiles are shown for: (**A**) Canonical isoform (Q17921-1), (**B**) Isoform-2 (Q17921-2), (**C**) Isoform-3 (Q17921-3), (**D**) Isoform-4 (Q17921-4), (**E**) Isoform-5 (Q17921-5), (**F**) Isoform-6 (Q17921-6), (**G**) Isoform-7 (Q17921-7). Disorder propensities were predicted using four members of the PONDR family depicted as different colors.

Disorder profiles for PDB entry 3MT5 [57] shows intrinsic disorder between residues 340–342 (1–3), 571–576 (232–237), 614–675 (275–336), 808–816 (469–477), 834–868 (495–529), 946–948 (607–609), 1021–1024 (682–685), and 1057–1065 (718–726). Numbers in brackets were obtained by renumbering residues as 1–726 (Figure 5C). Furthermore, the computational analysis of disorder profiles of human and mouse BK channels reveals the conservation of intrinsic disorder predispositions in their amino acid sequences. We also analyzed how predicted disorder propensities are affected by alternative splicing, based on the observation of conservation. There are seven isoforms of human BK produced by alternative splicing. Isoform 1 (Q12791-1) is a canonical isoform known as SAKCA. Isoform 2 (Q12791-2) differs from the canonical isoform by possessing a substitution of residues PKMSIYKRMRRACCFDCGRSERDCSCMSGRVRGNVDTLERAFPLSSVSVNDCSTSFRAF (698–756) for just one residue, L698, and by substituting the L828 of the canonical form with the sequence LVTGWMPYLGPRVLMTCLDIGVVCMPTDIQSTSPASIKKFKE. Isoform 3 (Q12791-3) differs from the canonical isoform, possessing a substitution of residue L643 with a sequence RSRKR. Isoform 4 (Q12791-4) differs from the canonical isoform by possessing a substitution of residues 698–756 for a shorter segment LKVAARSRYSKDPFEFKKETPNSRLVTEPV. Isoform 5 (Q12791-5) differs from the canonical isoform by possessing a substitution of residues 698–756 for a single residue L698. Isoform 6 (Q12791-6) differs from the canonical isoform by possessing a substitution of residues in region 127–168 and missing region 169–1236. Finally, isoform 7 (Q12791-7) possesses changes within the 698–756 region. Analyses of IDR in these isoforms shows that most of the human BK channel regions affected by alternative splicing are predicted as disordered (Figure 6).

**Figure 7:**
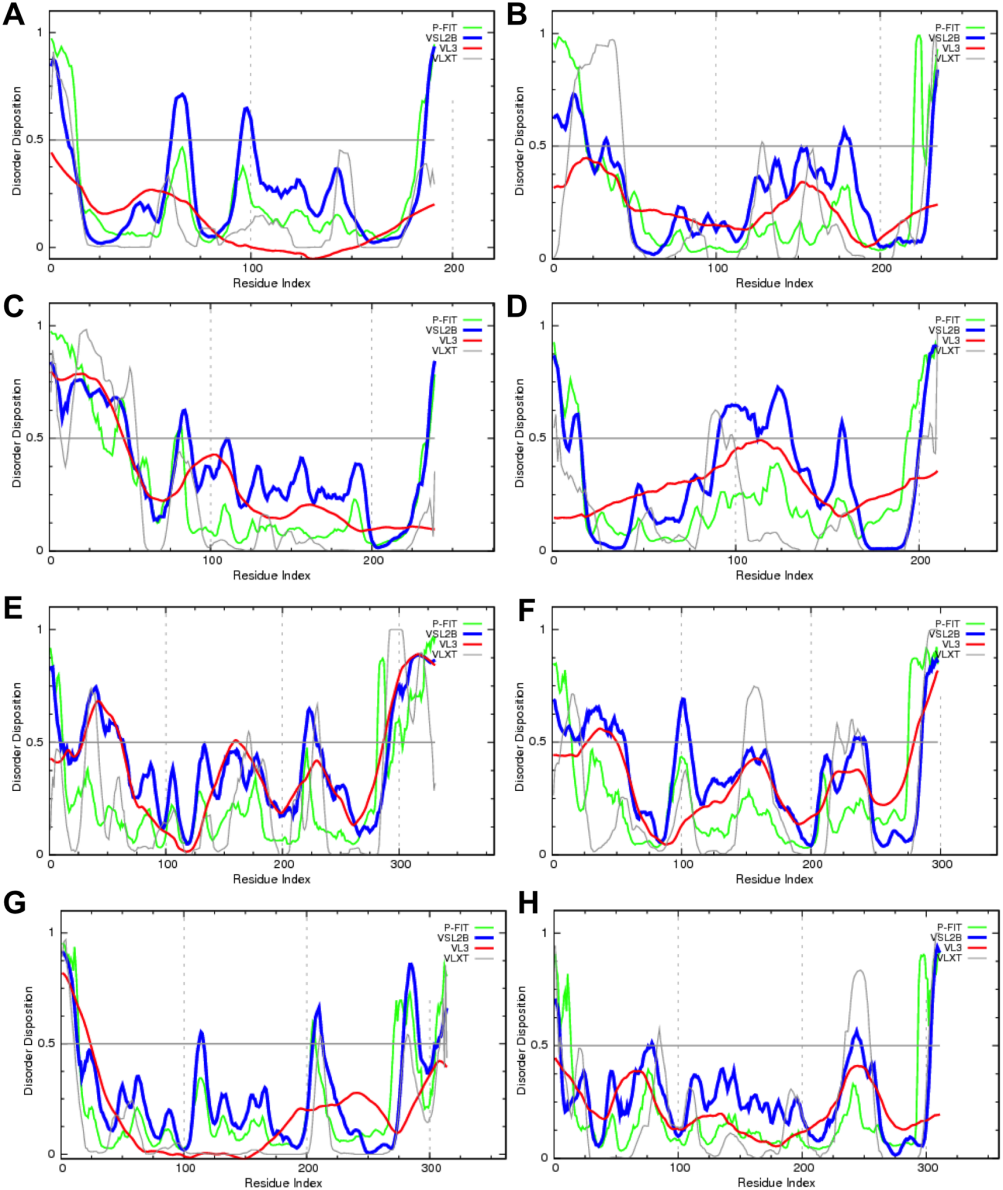
Intrinsic disorder profiles for BK accessory proteins. Disorder profiles are shown for β-subunits (**A**) KCNMB1 (Q8CAE3), (**B**) KCNMB2 (Q9CZM9), (**C**) KCNMB3 (E9Q7U0), and (**D**) KCNMB4 (Q9JIN6), and γ-subunits (**E**) LRRC26 (Q91W20), (**F**) LRRC38 (A2VDH3), (**G**) LRRC52 (Q5M8M9), and (**H**) LRRC55 (Q3UY51). Disorder propensities were evaluated using four members of the PONDR family.

Among the obvious BKAPs are BK accessory β- and γ-subunits. These subunits interact with the BK channel via its S0 transmembrane region and consist of β-subunits KCNMB1 (Q8CAE3), KCNMB2 (Q9CZM9), KCNMB3 (E9Q7U0), and KCNMB4 (Q9JIN6), and γ-subunits LRRC26 (Q91W20), LRRC38 (A2VDH3), LRRC52 (Q5M8M9), and LRRC55 (Q3UY51). Figure 7 represents disorder profiles evaluated for these eight accessory proteins by a family of PONDR predictors. The results show that all of these proteins possess various amounts of IDPRs.

**Figure 8:**
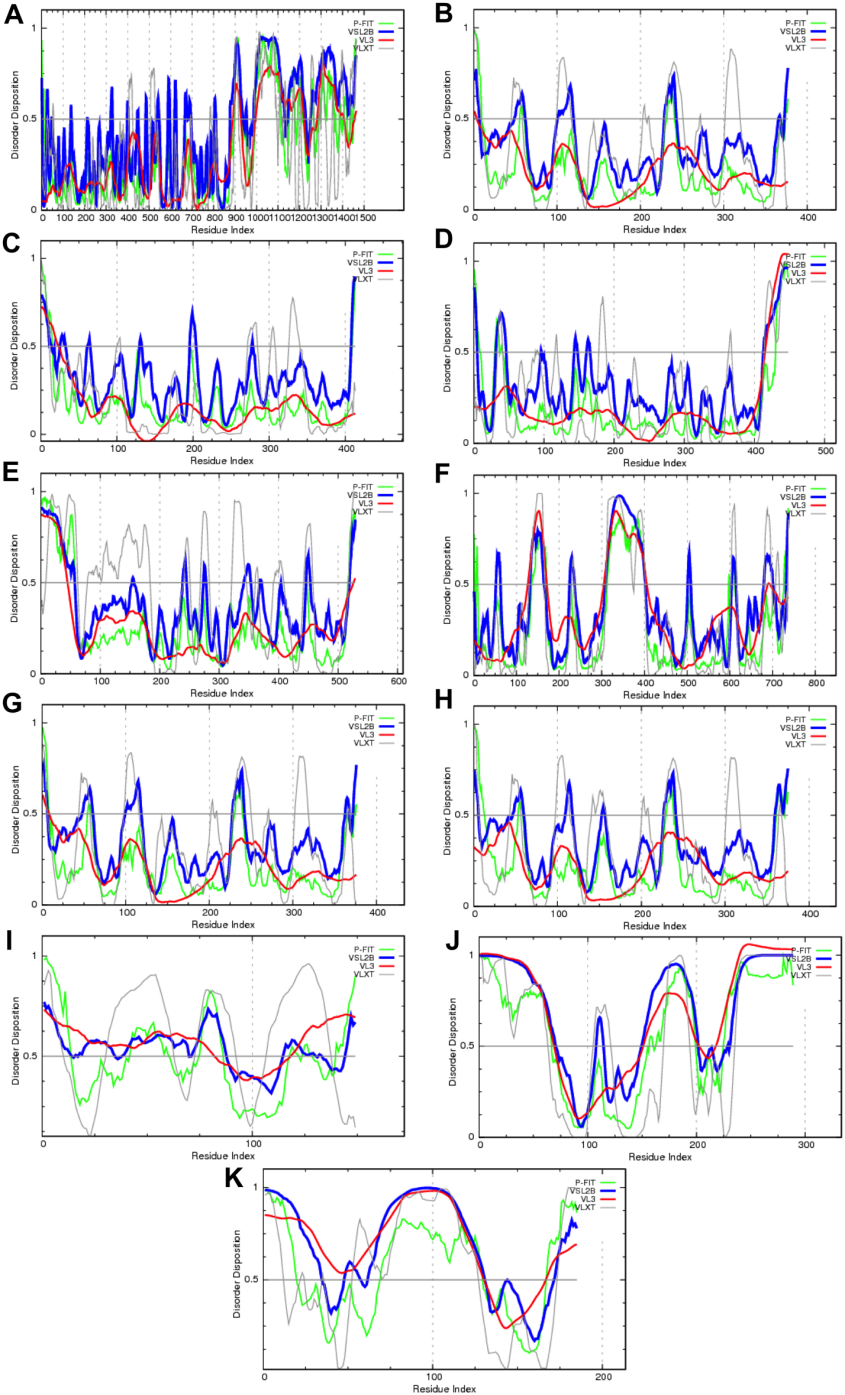
Intrinsic disorder profiles for hub proteins of the BK interactome. Disorder profiles are shown for: (**A**) NMDA receptor (P35436), (**B**) α-actin (P68134), (**C**) Aspartate aminotransferase (P05201), (**D**) α-tubulin (P68368), (**E**) ATP synthase beta subunit (P56480), (**F**) protein kinase C epsilon (P16054), (**G**) γ-actin (P63268), (**H**) β-actin (P60710), (**I**) calmodulin (P62204), (**J**) Protein SET (Q9EQU5), (**K**) and chromobox homolog 1 (P83917). Disorder propensities were evaluated using four members of the PONDR family.

Some of the BKAPs serve as hubs of the BK interactome, as they are involved in interactions with numerous binding partners [22], [23]. Figure 8 represents the peculiarities of the disorder distribution in 11 hub proteins, such as the NMDA receptor (20 interactions within the BK interactome), α-actin (19 interactions), aspartate aminotransferase (15 interactions), α-tubulin (15 interactions), ATP synthase beta subunit (ATP5b, 14 interactions), protein kinase c epsilon (11 interactions), γ-actin (9 interactions), β-actin (8 interactions), calmodulin (6 interactions), protein SET (6 interactions), and chromobox homolog 1 (6 interactions). This analysis revealed that these hub proteins possess various amounts of disorder and some of them (e.g., protein SET and chromobox homolog 1) are predicted as mostly disordered. These hub proteins are predicted to have numerous disorder-based interactions sites, MoRFs and AIBSs (see Table 3).

**Table 3:** The extent of disorder-based interaction sites (MoRFs and AIBSs) in hub proteins of the BK interactome.

Protein (UniProt ID)	Number of AIBSs	AIBS location	Number of MoRFs	MoRF location
Glutamate receptor ionotropic, NMDA (P35436)	20	927–932, 942, 966–968, 972–986, 998–1000, 1012–1028, 1044–1065, 1086–1107, 1115–1121, 1130–1147, 1182–1193, 1217–1224, 1234–1247, 1278–1281, 1322–1333, 1336–1337, 1375–1379, 1400–1407, 1423–1429, 1448–1453	35	9–16, 37–41, 67–72, 199–205, 222–226, 249–260, 281–286, 333–345, 358–367, 409–417, 473–480, 578–583, 602–614, 632–640, 692–705, 729–735, 775–780, 792–798, 816–821, 836–847, 864–874, 896–904, 925–931, 942–961, 970–982, 1008–1021, 1045–1058, 1097–1105, 1130–1154, 1184–1194, 1286–1301, 1321–1331, 1360–1375, 1422–1430, 1447–1464
Actin, α skeletal muscle (P68134)	1	376–377	9	11–17, 71–76, 90–94, 139–142, 222–226, 243–255, 261–264, 353–360, 372–377
Aspartate aminotransferase, cytoplasmic (P05201)	1	19–20	14	10–25, 44–56, 116–125, 155–163, 182–194, 210–226, 238–248, 264–270, 287–292, 310–320, 332–339, 355–368, 373–380, 405–413
Tubulin α-4A chain (P68368)	2	20–25, 51–54	8	19–28, 46–58, 86–90, 132–140, 141–159, 313–342, 401–409, 422–438
ATP synthase subunit β (P56480)	2	37–39, 339–341	12	27–38, 61–73, 139–150, 176–180, 186–193, 214–222, 250–256, 285–292, 303–310, 356–364, 388–396, 521–529
Protein kinase C epsilon type (P16054)	5	126–130, 298–308, 323–326, 352–371,406–413	17	7–13, 36–47, 125–133, 212–222, 300–311, 317–326, 328–341, 345–363, 371–383, 401–419, 436–441, 497–500, 524–540, 590–603, 620–633, 666–678, 724–737
Actin, γ-enteric smooth m. (P63268)	0		5	10–17, 88–94, 221–225, 242–263, 370–376
Actin, cytoplasmic 1 (P60710)	1	374–375	5	5–16, 84–93, 220–224, 241–262, 369–375
Calmodulin (P62204)	5	10–21, 64–73, 90–93, 100–106, 139–146	5	9–20, 32–38, 67–75, 87–96, 140–149
Protein SET (Q9EQU5)	5	1–14, 20–29, 92–94, 134–141, 199–289	8	3–13, 38–56, 139–142, 171–178, 197–210, 225–238, 251–266, 280–289
Chromobox protein homolog 1 (P83917)	4	33–45, 56–71, 115–140, 152–169	5	15–29, 32–46, 62–73, 131–141, 163–171

Hub proteins are defined as proteins involved in at least 6 protein-protein interactions.

## Discussion

This is the first study to examine peculiarities of intrinsic disorder propensity in the large conductance calcium-activated K^+^ channel and its BKAPs. Several recent studies explored IDPRs in other channel types or their subunits, including the K^+^ voltage-gated channel [16], [64], the β-subunit of BK [65], and the NMDA receptor [66]. These IDPRs were defined mechanistically in relation to channel activity or interactions with cytoskeletal proteins. Recent proteomic and functional data suggest, however, that BK interacts with a number of proteins that are related to cellular homeostasis. In addition to interactions with cytoskeletal and membrane proteins, BK interacts with signaling proteins involved with apoptosis/survival, such as Akt [22], [23], GSK [67], and annexin 5 [23]. Given these data and the ubiquity of this channel, a thorough evaluation was warranted relative to known BK crystallography data and the underlying mechanisms regulating BK interactions.

### IDRs and BK Crystallography

Presently, crystal structures exist for the C-terminal intracellular domain of the highly homologous human BK α-1 subunit (residues 393–1178). These are represented by a tetrameric complex of human BK intracellular subunits known as the intracellular gating ring [68] and a monomeric form of this domain [63]. Our results emphasize, here, that although the crystal structure of the C-terminal domain was determined, both structures contain multiple regions of missing electron density. These regions correspond to highly dynamic regions with very prominent conformational flexibility. Curiously, our data show that all these regions of missing electron density coincide or overlap with regions predicted as disordered (i.e., possess disorder scores above 0.5) or flexible (with disorder scores close to 0.5), as demonstrated for human BK and its monomeric form [63]. We evaluated these disorder propensities using members of the PONDR family of intrinsic disorder predictors. Here, scores above 0.5 correspond to disordered residues/regions. Furthermore, our comparisons suggest that the distribution of disorder within the amino acid sequences are conserved for human and mouse BK channels. Thus, we determined how predicted disorder propensities are affected by alternative splicing in the seven known BK isomers.

Previous studies reveal a strong correlation between alternative splicing and IDPRs [69]. Our data show that most of the human BK regions affected by alternative splicing are predicted as disordered, using four members of the PONDR family. This finding has important functional and structural implications. Alternative splicing enables functional and regulatory diversity, while its ID avoids the structural complications associated with the deletion of portions of well-folded proteins [69]. Moreover, studies reveal that protein segments produced by alternative splicing often contain IDRs that are enriched in posttranslational modification sites and embedded conserved binding motifs [70]. Alternative splicing of IDRs, containing linear interaction motifs and/or post-translational modification sites, results in complete rewiring of protein interactions [71]. This finding is underscored by our data showing that the IDRs in BK are rich in EMLs and MoRFs.

### IDPRs and MoRFs Vary in BK C-term Variants

While there are similarities between the different BK variants regarding disordered regions, an examination of the three C-terminal regions shows differences between ERL and VYR versus the longer DEC type. These differences at the protein C-termini may regulate placement of various BK variants in different subcellular regions [72]. ERL and VYR variants show IDPRs approximately between residues 630 and 750 and a small region at the C-terminal end. In contrast, the disordered region in the DEC type variant was longest at the very C-terminus, between residues 1085 and 1200. The IDPR at the end of the long cytoplasmic tail in all three variants probably allows this region to unfold and sample larger volumes in the cytoplasm. Since this is the tail end of the polypeptide, the idea of “fly-casting” [73] is especially relevant, as this C-terminal tail, particularly for the DEC variant where the C-term is especially long, contains many EMLs. Thus, this peptide string is ripe for protein-protein interactions. Residues 630–750 encompass the linker region connecting the two RCK domains, which form a gating ring and confer sensitivity to calcium [74]. This linker is unstructured, as it lacks electron density, in the most recent crystallography data of the RCK domains, using the monomeric form of human BK [74]. The linker, however, is functionally nonessential, since mutations in this region still result in the expression of wild type channels [74]. Rather, the length of the linker is important, as a decrease causes a shift to more positive conductance-voltage relationships [75]. In comparison, the BK DEC type lacked this long disordered linker region, bringing into question the flexibility of this variant between the two RCK regions.

A second disordered region is found within RCK2 in all three BK variants. The DEC type channel contains this IDPR approximately between residues 850–870. This sequence partially corresponds to the region between αQ and βO (residues 841–872) defined by Lee *et al*. [74]. Mutations within this short disordered region did not affect function. However, since this IDPR as well as the long linker contain an SH3 binding consensus sequence [76], this region is flexible and may expand as demonstrated for the C-terminus of NMDARs [66]. Such expansion would open these sites to protein-protein interactions, which is a likely scenario, given the preponderance of the many EMLs found in this region. In contrast, the ERL and VYR variants differ, since no disorder was determined in this region, but their corresponding IDPR is placed rather between residues 905 and 935. This shift in the second IDPR of these two variants is likely the result of the insertion site found in their longer linker region, but is absent in the DEC variant.

All types of the mouse BK channels analyzed in this study contain an important N-terminal disordered region (residues 58–95) that is located within the S0–S1 linker (**BK**-IS1, residues 43–113). Topologically, this region is located intracellularly, together with the Ca^2+^-sensing, large C-terminal intracellular ligand-binding part of the channel, accounting for two-thirds of the full channel. It is likely that flexibility of the S0–S1 linker is crucial for the decoupling of functional activities of two transmembrane regions of the BK channel. In fact, S0 provides a binding site for the β-subunit of the channels, whereas the S1–S4 transmembrane region of BK channels forms a voltage sensing domain. On the other hand, some studies indicate that S0 is located in close proximity to several voltage-sensing segments, such as S3 and S4, and can modulate the function of the voltage sensor, and therefore should be considered a part of the voltage-sensor domain [77]–[80]. It was also suggested that this intracellular **BK**-IS1 segment is essential for the Mg^2+^-dependent activation of **BK** **channel** function, with Asp99 of this segment, together with Asn172 of the voltage sensor domain and Glu374 and Glu399 of the cytosolic domain, being responsible for Mg^2+^ coordination [81], [82]. Recently, multidimensional heteronuclear NMR analysis of this region in the presence of dodecylphosphocholine (DPC) micelles revealed that **BK**-IS1 contains two amphipathic α-helices, which are stabilized through interaction with a membrane and are connected by a very flexible 36-residue loop (residues 57–92) [83]. This observation is in great agreement with our computational analysis that predicts disorder between residues 58–95 of the **BK**-IS1 region.

Interestingly, whereas the γ-MoRF type dominated the BK variants, the BK channels varied in the distribution of MoRFs within their C-terminal regions. Both ERL and VYR variants contained MoRF regions in the long linker as well as at the C-terminal end. The DEC variant possessed MoRF regions between residues 661–721, in the absence of a long IDPR, and also at the disordered C-terminal end. Since long disordered regions are defined as segments of at least 30 disordered residues, the former is likely the result of a lack of an insertion site and fragmented disordered regions that contain IDPRs shorter than 30 residues connected by short ordered regions.

### β and γ Subunits and other BKAPs Show Extensive, Functionally Important Disorder

The cytosolic N-terminal segments of auxiliary β-subunits [84] inactivate the BK channel. Inactivation occurs in two steps, an initial transition to a pre-inactivated conducting state followed by transition to a non-conducting inactivated state [85]. An effective inactivator of BK channels is the intrinsically disordered N-terminal peptide derived from β3a-subunit residues 1–55 [65]. The intrinsically disordered nature of this segment was established by circular dichroism spectroscopy and solution NMR analysis [65]. In agreement with these earlier observations, our results show that the N-terminal region of the KCNMB3 (β3a-subunit) is predicted as mostly disordered. Importantly, the N-terminal tails of the other β-subunits are able to block the BK channel and are predicted as disordered, suggesting that intrinsic disorder may play a role in the function of these important modulators of BK activity. Similarly, γ-subunits, which are leucine-rich repeat (LRR)-containing membrane proteins, are required for the conversion of BK from a high-voltage to a low-voltage activated type channel in non-excitable cells [86]. Although these γ-subunits are structurally and functionally distinct from β-subunits [87], they are predicted to possess substantial amounts of intrinsic disorder.

Our global disorder analysis revealed that many BKAPs possess numerous short IDPRs, while others were completely disordered. Both cytoplasmic and membrane/cytoskeletal BKAPs contained a relatively equal distribution of MoRFs that form α-helices upon binding (α-MoRFs), and those that form coils (γ-MoRFs), suggesting both proline scarce and rich partners, respectively. Those exhibiting regions with MoRFs likely play a role as a signaling protein [38].

Within the population of BKAPs are a number of hub proteins [22], [23]. Since these hub proteins are involved in promiscuous interactions with multiple partners, they contribute extensively to the BK interactome. All of these hubs showed extensive disorder, and were enriched in MoRF sites and AIBSs. Hub proteins are reported to have a greater number of disordered regions than non-hubs [13], and likely have fewer loops/coils too [88]. Hub proteins from the BK interactome, such as NMDAR, show extensive disorder, suggesting the potential for interacting with numerous proteins. Interestingly, some proteins such as GSK3β have different regions of disorder that are independently involved in different regulatory pathways [13]. Similar studies are needed to determine such independence in BK hubs, given the potential ramifications to BK expression and function.

There is an extensive amount of data supporting the abundance and biological roles of intrinsic disorder in several hubs of the BK interactome. The crucial role of intrinsic disorder in structure, function, and folding of actin was discussed in a recent comprehensive review [89]. Here, it was pointed out that actin preserves mobility even when complexed with an actin-binding protein and crystallized. This is evident from the presence of regions with relatively high values of the B-factor, which reflects the relative vibrational motion of different structural parts. Furthermore, in its complex with the chimera of gelsolin domain 1 and the C-terminal domain of thymosin β-4 (PDB ID: 1T44) [90], actin has two regions of missing electron density (residues 25–27 and 39–50), corresponding to highly mobile segments that are disordered. Another example is given by calmodulin, a 148-residue long intracellular protein involved in numerous eukaryotic regulatory processes and highly conserved throughout the eukaryotic kingdom. Calmodulin has diverse modes of interaction with numerous binding partners, as it binds short peptides and proteins in the presence or absence of Ca^2+^, reversibly and irreversibly, and as an inhibitor or an activator [91]. Structural studies show that calmodulin interactions with binding targets (CaMBTs) involve disorder-to-order transitions. Here, the flexible linker of calmodulin becomes structured upon complex formation, allowing the globular domains to wrap around the CaMBT [92]–[94]. Furthermore, data indicate that several CaMBTs are disordered before association with calmodulin, a hypothesis generalized and extended to all CaMBTs, based on detailed computational analyses [94]. In fact, many, if not all, clamodulin-binding targets are intrinsically disordered. These sites can be predicted by a specialized predictor that utilizes intrinsic disorder propensity of calmodulin binding motifs and flanking regions [94].

In conclusion, BK and BKAPs show a propensity for intrinsic disorder. In the BK channel, many of these regions lay between the RCK domains that are relevant for BK function, thereby allowing for folding flexibility that is contingent on the binding of protein partners. Thus, for the future, it is of interest to determine which partners bind in these regions and how they affect BK function. BKAPs including β and γ subunits also show an abundance of functionally important intrinsic disorder.

## Supporting Information

Table S1 Aligned ELM annotations for BK channel variants. Grouping of 59 ELM classes into 4 types including cleavage sites (CLV), ligand binding sites (LIG), sites of posttranslational modification (MOD) and subcellular targeting sites (TRG).
(PDF)

Table S2 Aligned ClustalW annotations for overall characterization of BK channel variants. Sequence profiles of BK channel variants showing ID, MoRF regions, globular domains, and the four types of ELMs.
(PDF)

Table S3 Raw sequence data aligned for overall characterization of BK channel variants. Raw sequence data alignments of all 22 BK channel variants showing results of analyses for ID, MoRF regions, globular domains, and the four types of ELMs.
(DOC)

Table S4 Raw data for evolutionary conservation analysis. Conservation profiles for 22 BK channel variants calculated from the WOP profiles generated by PSI-BLAST.
(XLSX)
