# Supplementary material for: Intrinsic Disorder in the BK Channel and Its Interactome
Source: PLoS One. 2014 Apr 11;9(4):e94331. doi: 10.1371/journal.pone.0094331 (PMC3984161; doi:10.1371/journal.pone.0094331)
Supplement: Table S3 — Raw sequence data aligned for overall characterization of BK channel variants. Raw sequence data alignments of all 22 BK channel variants showing results of analyses for ID, MoRF regions, globular domains, and the four types of ELMs. (DOC) [file pone.0094331.s003.doc]

# Sequence alignment of BK channel variants

E3VRZ5 MDALIIPVTMEVPCDSRGQRMWWAFLASSMVTFFGGLFIILLWRTLKYLWTVCCHCGGKTKEAQKINNGSSQADGTLKPVDEKEEVVAAEVGWMTSVKDWAGVMISAQTLTGRVLVVLVFALSIGALVIYFIDSSNPIESCQNFYKDFTLQIDMAFNVFF

E3VRZ6 MDALIIPVTMEVPCDSRGQRMWWAFLASSMVTFFGGLFIVLLWRTLKYLWTVCCHCGGKTKEAQKINNGSSQADGTLKPVDEKEEVVAAEVGWMTSVKDWAGVMISAQTLTGRVLVVLVFALSIGALVIYFIDSSNPIESCQNFYKDFTLQIDMAFNVFF

E3VRZ4 MDALIIPVTMEVPCDSRGQRMWWAFLASSMVTFFGGLFIILLWRTLKYLWTVCCHCGGKTKEAQKINNGSSQADGTLKPVDEKEEVVAAEVGWMTSVKDWAGVMISAQTLTGRVLVVLVFALSIGALVIYFIDSSNPIESCQNFYKDFTLQIDMAFNVFF

E3VRY6 MDALIIPVTMEVPCDSRGQRMWWAFLASSMVTFFGGLFIILLWRTLKYLWTVCCHCGGKTKEAQKINNGSSQADGTLKPVDEKEEVVAAEVGWMTSVKDWAGVMISAQTLTGRVLVVLVFALSIGALVIYFIDSSNPIESCQNFYKDFTLQIDMAFNVFF

E3VRZ2 MDALIIPVTMEVPCDSRGQRMWWAFLASSMVTFFGGLFIILLWRTLKYLWTVCCHCGGKTKEAQKINNGSSQADGTLKPVDEKEEVVAAEVGWMTSVKDWAGVMISAQTLTGRVLVVLVFALSIGALVIYFIDSSNPIESCQNFYKDFTLQIDMAFNVFF

E3VRY7 MDALIIPVTMEVPCDSRGQRMWWAFLASSMVTFFGGLFIILLWRTLKYLWTVCCHCGGKTKEAQKINNGSSQADGTLKPVDEKEEVVAAEVGWMTSVKDWAGVMISAQTLTGRVLVVLVFALSIGALVIYFIDSSNPIESCQNFYKDFTLQIDMAFNVFF

E3VRZ7 MDALIIPVTMEVPCDSRGQRMWWAFLASSMVTFFGGLFIILLWRTLKYLWTVCCHCGGKTKEAQKINNGSSQADGTLKPVDEKEEVVAAEVGWMTSVKDWAGVMISAQTLTGRVLVVLVFALSIGALVIYFIDSSNPIESCQNFYKDFTLQIDMAFNVFF

E3VRZ8 MDALIIPVTLEVPCDSRGQRMWWAFLASSMVTFFGGLFIILLWRTLKYLWTVCCHCGGKTKEAQKINNGSSQADGTLKPVDEKEEVVAAEVGWMTSVKDWAGVMISAQTLTGRVLVVLVFALSIGALVIYFIDSSNPIESCQNFYKDFTLQIDMAFNVFF

E3VRZ9 MDALIIPVTMEVPCDSRGQRMWWAFLASSMVTFFGGLFIILLWRTLKYLWTVCCHCGGKTKEAQKINNGSSQADGTLKPVDEKEEVVAAEVGWMTSVKDWAGVMISAQTLTGRVLVVLVFALSIGALVIYFIDSSNPIESCQNFYKDFTLQIDMAFNVFF

E3VRZ3 MDALIIPVTMEVPCDSRGQRMWWAFLASSMVTFFGGLFIILLWRTLKYLWTVCCHCGGKTKEAQKINNGSSQADGTLKPVDEKEEVVAAEVGWMTSVKDWAGVMISAQTLTGRVLVVLVFALSIGALVIYFIDSSNPIESCQNFYKDFTLQIDMAFNVFF

E3VS03 MDALIIPVTMEVPCDSRGQRMWWAFLASSMVTFFGGLFIILLWRTLKYLWTVCCHCGGKTKEAQKINNGSSQADGTLKPVDEKEEVVAAEVGWMTSAKDWAGVMISAQTLTGRVLVVLVFALSIGALVIYFIDSSNPIESCQNFYKDFTLQIDMAFNVFF

E3VS04 MDALIIPVTMEVPCDSRGQRMWWAFLASSMVTFFGGLFIILLWRTLKYLWTVCCHCGGKTKEAQKINNGSSQADGTLKPVDEKEEVVAAEVGWMTSVKDWAGVMISAQTLTGRVLVVLVFALSIGALVIYFIDSSNPIESCQNFYKDFTLQIDMAFNVFF

E3VS00 MDALIIPVTMEVPCDSRGQRMWWAFLASSMVTFFGGLFIILLWRTLKYLWTVCCHCGGKTKEAQKINNGSSQADGTLKPVDEKEEVVAAEVGWMTSVKDWAGVMISAQTLTGRVLVVLVFALSIGALVIYFIDSSNPIESCQNFYKDFTLQIDMAFNVFF

E3VS01 MDALIIPVTMEVPCDSRGQRMWWAFLASSMVTFFGGLFIILLWRTLKYLWTVCCHCGGKTKEAQKINNGSSQADGTLKPVDEKEEVVAAEVGWMTSVKDWAGVMISAQTLTGRVLVVLVFALSIGALVIYFIDSSNPIESCQNFYKDFTLQIDMAFNVFF

E3VRY4 MDALIIPVTMEVPCDSRGQRMWWAFLASSMVTFFGGLFIILLWRTLKYLWTVCCHCGGKTKEAQKINNGSSQADGTLKPVDEKEEVVAAEVGWMTSVKDWAGVMISAQTLTGRVLVVLVFALSIGALVIYFIDSSNPIESCQNFYKDFTLQIDMAFNVFF

E3VS02 MDALIIPVTMEVPCDSRGQRMWWAFLASSMVTFFGGLFIILLWRTLKYLWTVCCHCGGKTKEAQKINNGSSQADGTLKPVDEKEEVVAAEVGWMTSVKDWAGVMISAQTLTGRVLVVLVFALSIGALVIYFIDSSNPIESCQNFYKDFTLQIDMAFNVFF

E3VRY5 MDALIIPVTMEVPCDSRGQRMWWAFLASSMVTFFGGLFIILLWRTLKYLWTVCCHCGGKTK------------------------------------------------------VVLVFALSIGALVIYFIDSSNPIESCQNFYKDFTLQIDMAFNVFF

E3VRZ0 MDALIIPVTMEVPCDSRGQRMWWAFLASSMVTFFGGLFIILLWRTLKYLWTVCCHCGGKTKEAQKINNGSSQADGTLKPVDGGEEVVAAEVGWMTSVKDWAGVMISAQTLTGRVLVVLVFALSIGALVIYFIDSSNPIESCQNFYKDFTLQIDMAFNVFF

E3VRY8 MDALIIPVTMEVPCDSRGQRMWWAFLASSMVTFFGGLFIILLWRTLKYLWTVCCHCGGKTKEAQKINNGSSQADGTLKPVDEKEEVVAAEVGWMTSVKDWAGVMISAQTLTGRVLVVLVFALSIGALVIYFIDSSNPIESCQNFYKDFTLQIDMAFNVFF

E3VRZ1 MDALIIPVTMEVPCDSRGQRMWWAFLASSMVTFFGGLFIILLWRTLKYLWTVCCHCGGKTKEAQKINNGSSQADGTLKPVDEKEEVVAAEVGWMTSVKDWAGVMISAQTLTGRVLVVLVFALSIGALVIYFIDSSNPIESCQNFYKDFTLQIDMAFNVFF

C3VLD3 MDALIIPVTMEVPCDSRGQRMWWAFLASSMVTFFGGLFIILLWRTLKYLWTVCCHCGGKTKEAQKINNGSSQADGTLKPVDEKEEVVAAEVGWMTSVKDWAGVMISAQTLTGRVLVVLVFALSIGALVIYFIDSSNPIESCQNFYKDFTLQIDMAFNVFF

E3VRY9 MDALIIPVTMEVPCDSRGQRMWWAFLASSMVTFFGGLFIILLWRTLKYLWTVCCHCGGKTKEAQKINNGSSQADGTLKPVDEKEEVVAAEVGWMTSVKDWAGVMISAQTLTGRVLVVLVFALSIGALVIYFIDSSNPIESCQNFYKDFTLQIDMAFNVFF

**ALIGN *********.*****************************.********************* ***********************************************

E3VRZ5 LLYFGLRFIAANDKLWFWLEVNSVVDFFTVPPVFVSVYLNRSWLGLRFLRALRLIQFSEILQFLNILKTSNSIKLVNLLSIFISTWLTAAGFIHLVENSGDPWENFQNNQALTYWECVYLLMVTMSTVGYGDVYAKTTLGRLFMVFFILGGLAMFASYVP

E3VRZ6 LLYFGLRFIAANDKLWFWLEVNSVVDFFTVPPVFVSVYLNRSWLGLRFLRALRLIQFSEILQFLNILKTSNSIKLVNLLSIFISTWLTAAGFIHLVENSGDPWENFQNNQALTYWECVYLLMVTMSTVGYGDVYAKTTLGRLFMVFFILGGLAMFASYVP

E3VRZ4 LLYFGLRFIAANDKLWFWLEVNSVVDFFTVPPVFVSVYLNRSWLGLRFLRALRLIQFSEILQFLNILKTSNSIKLVNLLSIFISTWLTAAGFIHLVENSGDPWENFQNNQALTYWECVYLLMVTMSTVGYGDVYAKTTLGRLFMVFFILGGLAMFASYVP

E3VRY6 LLYFGLRFIAANDKLWFWLEVNSVVDFFTVPPVFVSVYLNRSWLGLRFLRALRLIQFSEILQFLNILKTSNSIKLVNLLSIFISTWLTAAGFIHLVENSGDPWENFQNNQALTYWECVYLLMVTMSTVGYGDVYAKTTLGRLFMVFFILGGLAMFARYVP

E3VRZ2 LLYFGLRFIAANDKLWFWLEVNSVVDFFTVPPVFVSVYLNRSWLGLRFLRALRLIQFSEILQFLNILKTSNSIKLVNLLSIFISTWLTAAGFIHLVENSGDPWENFQNNQALTYWECVYLLMVTMSTVGYGDVYAKTTLGRLFMVFFILGGLAMFASYVP

E3VRY7 LLYFGLRFIAANDKLWFWLEVNSVVDFFTVPPVFVSVYLNRSWLGLRFLRALRLIQFSEILQFLNILKTSNSIKLVNLLSIFISTWLTAAGFIHLVENSGDPWENFQNNQALTYWECVYLLMVTMSTVGYGDVYAKTTLGRLFMVFFILGGLAMFASYVP

E3VRZ7 LLYFGLRFIAANDKLWFWLEVNSVVDFFTVPPVFVSVYLNRSWLGLRFLRALRLIQFSEILQFLNILKTSNSIKLVNLLSIFISTWLTAAGFIHLVENSGDPWENFQNNQALTYWECVYLLMVTMSTVGYGDVYAKTTLGRLFMAFFILGGLAMFASYVP

E3VRZ8 LLYFGLRFIAANDKLWFWLEVNSVVDFFTVPPVFVSVYLNRSWLGLRFLRALRLIQFSEILQFLNILKTSNSIKLVNLLSIFISTWLTAAGFIHLVENSGDPWENFQNNQALTYWECVYLLMVTMSTVGYGDVYAKTTLGRLFMVFFILGGLAMFASYVP

E3VRZ9 LLYFGLRFIAANDKLWFWLEVNSVVDFFTVPPVFVSVYLNRSWLGLRFLRALRLIQFSEILQFLNILKTSNSIKLVNLLSIFISTWLTAAGFIHLVENSGDPWENFQNNQALTYWECVYLLMVTMSTVGYGDVYAKTTLGRLFMVFFILGGLAMFASYVP

E3VRZ3 LLYFGLRFIAANDKLWFWLEVNSVVDFFTVPPVFVSVYLNRSWLGLRFLRALRLIQFSEILQFLNILKTSNSIKLVNLLSIFISTWLTAAGFIHLVENSGDPWENFQNNQALTYWECVYLLMVTMSTVGYGDVYAKTTLGRLFMVFFILGGLAMFASYVP

E3VS03 LLYFGLRFIAANDKLWFWLEVNSVVDFFTVPPVFVSVYLNRSWLGLRFLRALRLIQFSEILQFLNILKTSNSIKLVNLLSIFISTWLTAAGFIHLVENSGDPWENFQNNQALTYWECVYLLMVTMSTVGYGDVYAKTTLGRLFMVFFILGGLAMFASYVP

E3VS04 LLYFGLRFIAANDKLWFWLEVNSVVDFFTVPPVFVSVYLNRSWLGLRFLRALRLIQFSEILQFLNILKTSNSIKLVNLLSIFISTWLTAAGFIHLVENSGDPWENFQNNQALTYWECVYLLMVTMSTVGYGDVYAKTTLGRLFMVFFILGGLAMFASYVP

E3VS00 LLYFGLRFIAANDKLWFWLEVNSVVDFFTVPPVFVSVYLNRSWLGLRFLRALRLIQFSEILQFLNILKTSNSIKLVNLLSIFISTWLTAAGFIHLVENSGDPWENFQNNQALTYWECVYLLMVTMSTVGYGDVYAKTTLGRLFMVFFILGGLAMFASYVP

E3VS01 LLYFGLRFIAANDKLWFWLEVNSVVDFFTVPPVFVSVYLNRSWLGLRFLRALRLIQFSEILQFLNILKTSNSIKLVNLLPIFISTWLTAAGFIHLVENSGDPWENFQNNQALTYWECVYLLMVTMSTVGYGDVYAKTTLGRLFMVFFILGGLAMFASYVP

E3VRY4 LLYFGLRFIAANDKLWFWLEVNSVVDFFTVPPVFVSVYLNRSWLGLRFLRALRLIQFSEILQFLNILKTSNSIKLVNLLSIFISTWLTAAGFIHLVENSGDPWENFQNNQALTYWECVYLLMVTMSTVGYGDVYAKTTLGRLFMVFFILGGLAMFASYVP

E3VS02 LLYFGLRFIAANDKLWFWLEVNSVVDFFTVPPVFVPVYLNRSWLGLRFLRALRLIQFSEILQFLNILKTSNSIKLVNLLSIFISTWLTAAGFIHLVENSGDPWENFQNNQALTYWECVYLPMVTMSTVGYGDVYAKTTLGRLFMVFFILGGLAMFASYVP

E3VRY5 LLYFGLRFIAANDKLWFWLEVNSVVDFFTVPPVFVSVYLNRSWLGLRFLRALRLIQFSEILQFLNILKTSNSIKLVNLLSIFISTWLTAAGFIHLVENSGDPWENFQNNQALTYWECVYLLMVTMSTVGYGDVYAKTTLGRLFMVFFILGGLAMFASYVP

E3VRZ0 LLYFGLRFIAANDKLWFWLEVNSVVDFFTVPPVFVSVYLNRSWLGLRFLRALRLIQFSEILQFLNILKTSNSIKLVNLLSIFISTWLTAAGFIHLVENSGDPWENFQNNQALTYWECVYLLMVTMSTVGYGDVYAKTTLGRLFMAFFILGGLAMFASYVP

E3VRY8 LLYFGLRFIAANDKLWFWLEVNSVVDFFTVPPVFVSVYLNRSWLGLRFLRALRLIQFSEILQFLNILKTSNSIKLVNLLSIFISTWLTAAGFIHLVENSGDPWENFQNNQALTYWECVYLLMVTMSTVGYGDVYAKTTLGRLFMVFFILGGLAMFASYVP

E3VRZ1 LLYFGLRFIAANDKLWFWLEVNSVVDFFTVPPVFVSVYLNRSWLGLRFLRALRLIQFSEILQFLNILKTSNSIKLVNLLSIFISTWLTAAGFIHLVENSGDPWENFQNNQALTYWECVYLLMVTMSTVGYGDVYAKTTLGRLFMVFFILGGLAMFASYVP

C3VLD3 LLYFGLRFIAANDKLWFWLEVNSVVDFFTVPPVFVSVYLNRSWLGLRFLRALRLIQFSEILQFLNILKTSNSIKLVNLLSIFISTWLTAAGFIHLVENSGDPWENFQNNQALTYWECVYLLMVTMSTVGYGDVYAKTTLGRLFMVFFILGGLAMFASYVP

E3VRY9 LLYFGLRFIAANDKLWFWLEVNSVVDFFTVPPVFVSVYLNRSWLGLRFLRALRLIQFSEILQFLNILKTSNSIKLVNLLSIFISTWLTAAGFIHLVENSGDPWENFQNNQALTYWECVYLLMVTMSTVGYGDVYAKTTLGRLFMVFFILGGLAMFASYVP

**ALIGN ***********************************.*******************************************.**************************************** ***********************.*********** *****

E3VRZ5 EIIELIGNRKKYGGSYSAVSGRKHIVVCGHITLESVSNFLKDFLHKDRDDVNVEIVFLHNISPNLELEALFKRHFTQVEFYQGSVLNPHDLARVKIESADACLILANKYCADPDAEDASNIMRVISIKNYHPKIRIITQMLQYHNKAHLLNIPSWNWKEG

E3VRZ6 EIIELIGNRKKYGGSYSAVSGRKHIVVCGHITLESVSNFLKDFLHKDRDDVNVEIVFLHNISPNLELEALFKRHFTQVEFYQGSVLNPHDLARVKIESADACLILANKYCADPDAEDASNIMRVISIKNYHPKIRIITQMLQYHNKAHLLNIPSWNWKEG

E3VRZ4 EIIELIGNRKKYGGSYSAVSGRKHIVVCGHITLESVSNFLKDFLHKDRDDVNVEIVFLHNISPNLELEALFKRHFTQVEFYQGSVLNPHDLARVKIESADACLILANKYCADPDAEDASNIMRVISIKNYHPKIRIITQMLQYHNKAHLLNIPSWNWKEG

E3VRY6 EIAALILNRNKFGGTFNKHGGRKHIVVCGHITLESVSNFLKDFLHKDRDDVNVEIVFLHNISPNLELEALFKRHFTQVEFYQGSVLNPHDLARVKIESADACLILANKYCADPDAEDASNIMRVISIKNYHPKIRIITQMLQYHNKAHLLNIPSWNWKEG

E3VRZ2 EIIELIGNRKKYGGSYSAVSGRKHIVVCGHITLESVSNFLKDFLHKDRDDVNVEIVFLHNISPNLELEALFKRHFTQVEFYQGSVLNPHDLARVKIESADACLILANKYCADPDAEDASNIMRVISIKNYHPKIRIITQMLQYHNKAHLLNIPSWNWKEG

E3VRY7 EIIELIGNRKKYGGSYSAVSGRKHIVVCGHITLESVSNFLKDFLHKDRDDVNVEIVFLHNISPNLELEALFKRHFTQVEFYQGSVLNPHDLARVKIESADACLILANKYCADPDAEDASNIMRVISIKNYHPKIRIITQMLQYHNKAHLLNIPSWNWKEG

E3VRZ7 EIIELIGNRKKYGGSYSAVSGRKHIVVCGHITLESVSNFLKDFLHKDRDDVNVEIVFLHNISPNLELEALFKRHFTQVEFYQGSVLNPHDLARVKIESADACLILANKYCADPDAEDASNIMRVISIKNYHPKIRIITQMLQYHNKAHLLNIPSWNWKEG

E3VRZ8 EIIELIGNRKKYGGSYSAVSGRKHIVVCGHITLESVSNFLKDFLHKDRDDVNVEIVFLHNISPNLELEALFKRHFTQVEFYQGSVLNPHDLARVKIESADACLILANKYCADPDAEDASNIMRVISIKNYHPKIRIITQMLQYHNKAHLLNIPSWNWKEG

E3VRZ9 EIIELIGNRKKYGGSYSAVSGRKHIVVCGHITLESVSNFLKDFLHKDRDDVNVEIVFLRNISPNLELEALFKRHFTQVEFYQGSVLNPHDLARVKIESADACLILANKYCADPDAEDASNIMRVISIKNYHPKIRIITQMLQYHNKAHLLNIPSWNWKEG

E3VRZ3 EIIELIGNRKKYGGSYSAVSGRKHIVVCGHITLESVSNFLKDFLHKDRDDVNVEIVFLHNISPNLELEALFKRHFTQVEFYQGSVLNPHDLARVKIESADACLILANKYCADPDAEDASNIMRVISIKNYHPKIRIITQMLQYHNKAHLLNIPSWNWKEG

E3VS03 EIIELIGNRKKYGGSYSAVSGRKHIVVCGHITLESVSNFLKDFLHKDRDDVNVEIVFLHNISPNLELEALFKRHFTQVEFYQGSVLNPHDLARVKIESADACLILANKYCADPDAEDASNIMRVISIKNYHPKIRIITQMLQYHNKAHLLNIPSWNWKEG

E3VS04 EIIELIGNRKKYGGSYSAVSGRKHIVVCGHITLESVSNFLKDFLHKDRDDVNVEIVFLHNISPNLELEALFKRHFTQVEFYQGSVLNPHDLARVKIESADACLILANKYCADPDAEDASNIMRVISIKNYHPKIRIITQMLQYHNKAHLLNIPSWNWKEG

E3VS00 EIIELIGNRKKYGGSYSAVSGRKHIVVCGHITLESVSNFLKDFLHKDRDDVNVEIVFLHNISPNLELEALFKRHFTQVEFYQGSVLNPHDLARVKIESADACLILANKYCADPDAEDASNIMRVISIKNYHPKIRIITQMLQYHNKAHLLNIPSWNWKEG

E3VS01 EIIELIGNRKKYGGSYSAVSGRKHIVVCGHITLESVSNFLKDFLHKDRDDVNVEIVFLHNISPNLELEALFKRHFTQVEFYQGSVLNPHDLARVKIESADACLILANKYCADPDAEDASNIMRVISIKNYHPKIRIITQMLQYHNKAHLLNIPSWNWKEG

E3VRY4 EIIELIGNRKKYGGSYSAVSGRKHIVVCGHITLESVSNFLKDFLHKDRDDVNVEIVFLHNISPNLELEALFKRHFTQVEFYQGSVLNPHDLARVKMESADACLILANKYCADPDAEDASNIMRVISIKNYHPKIRIITQMLQYHNKAHLLNIPSWNWKEG

E3VS02 EIIELIGNRKKYGGSYSAVSGRKHIVVCGHITLESVSNFLKDFLHKDRDDVNVEIVFLHNISPNLELEALFKRHFTQVEFYQGSVLNPHDLARVKIESADACLILANKYCADPDAEDASNIMRVISIKNYHPKIRIITQMLQYHNKAHLLNIPSWNWKEG

E3VRY5 EIIELIGNRKKYGGSYSAVSGRKHIVVCGHITLESVSNFLKDFLHKDRDDVNVEIVFLHNISPNLELEALFKRHFTQVEFYQGSVLNPHDLARVKIESADACLILANKYCADPDAEDASNIMRVISIKNYHPKIRIITQMLQYHNKAHLLNIPSWNWKEG

E3VRZ0 EIIELIGNRKKYGGSYSAVSGRKHIVVCGHITLESVSNFLKDFLHKDRDDVNVEIVFLHNISPNLELEALFKRHFTQVEFYQSSVLNPHDLARVKIESADACLILANKYCADPDAEDASNIMRVISIKNYHPKIRIITQMLQYHNKAHLLNIPSWNWKEG

E3VRY8 EIIELIGNRKKYGGSYSAVSGRKHIVVCGHITLESVSNFLKDFLHKDRDDVNVEIVFLHNISPNLELEALFKRHFTQVEFYQGSVLNPHDLARVKIESADACLILANKYCADPDAEDASNIMRVISIKNYHPKIRIITQMLQYHNKAHLLNIPSWNWKEG

E3VRZ1 EIIELIGNRKKYGGSYSAVSGRKHIVVCGHITLESVSNFLKDFLHKDRDDVNVEIVFLHNISPNLELEALFKRHFTQVEFYQGSVLNPHDLARVKIESADACLILANKYCADPDAEDASNIMRVISIKNYHPKIRIITQMLQYHNKAHLLNIPSWNWKEG

C3VLD3 EIIELIGNRKKYGGSYSAVSGRKHIVVCGHITLESVSNFLKDFLHKDRDDVNVEIVFLHNISPNLELEALFKRHFTQVEFYQGSVLNPHDLARVKIESADACLILANKYCADPDAEDASNIMRVISIKNYHPKIRIITQMLQYHNKAHLLNIPSWNWKEG

E3VRY9 EIIELIGNRKKYGGSYSAVSGRKHIVVCGHITLESVSNFLKDFLHKDRDDVNVEIVFLHNISPNLELEALFKRHFTQVEFYQGSVLNPHDLARVKIESADACLILANKYCADPDAEDASNIMRVISIKNYHPKIRIITQMLQYHNKAHLLNIPSWNWKEG

**ALIGN ** ** **.*.**... .**************************************.***********************.************.******************************************************************

E3VRZ5 DDAICLAELKLGFIAQSCLAQGLSTMLANLFSMRSFIKIEEDTWQKYYLEGVSNEMYTEYLSSAFVGLSFPTVCELCFVKLKLLMIAIEYKSANRESR----ILINPGNHLKIQEGTLGFFIASDAKEVKRAFFYCKACHDDVTDPKRIKK--CGCRRL-

E3VRZ6 DDAICLAELKLGFIAQSCLAQGLSTMLANLFSMRSFIKIEEDTWQKYYLEGVSNEMYTEYLSSAFVGLSFPTVCELCFVKLKLLMIAIEYKSANRESR----ILINPGNHLKIQEGTLGFFIASDAKEVKRAFFYCKACHDDVTDPKRIKK--CGCRRLK

E3VRZ4 DDAICLAELKLGFIAQSCLAQGLSTMLANLFSMRSFIKIEEDTWQKYYLEGVSNEMYTEYLSSAFVGLSFPTVCELCFVKLKLLMIAIEYKSANRESR----ILINPGNHLKIQEGTLGFFIASDAKEVKRAFFYCKACHDDVTDPKRIKK--CGCRRL-

E3VRY6 DDAICLAELKLGFIAQSCLAQGLSTMLANLFSMRSFIKIEEDTWQKYYLEGVSNEMYTEYLSSAFVGLSFPTVCELCFVKLKLLMIAIEYKSANRESR----ILINPGNHLKIQEGTLGFFIASDAKEVKRAFFYCKACHDDVTDPKRIKK--CGCRRLE

E3VRZ2 DDAICLAELKLGFIAQSCLAQGLSTMLANLFSMRSFIKIEEDTWQKYYLEGVSNEMYTEYLSSAFVGLSFPTVCELCFVKLKLLMIAIEYKSANRESRSRKRILINPGNHLKIQEGTLGFFIASDAKEVKRAFFTARPVMMTSQIPKELKNVAAGGSRLK

E3VRY7 DDAICLAELKLGFIAQSCLAQGLSTMLANLFSMRSFIKIEEDTWQKYYLEGVSNEMYTEYLSSAFVGLSFPTVCELCFVKLKLLMIAIEYKSANRESR----ILINPGNHLKIQEGTLGFFIASDAKEVKRAFFYCKACHDDVTDPKRIKK--CGCRRLE

E3VRZ7 DDAICLAELKLGFIAQSCLAQGLSTMLANLFSMRSFIKIEEDTWQKYYLEGVSNEMYTEYLSSAFVGLSFPTVCELCFVKLKLLMIAIEYKSANRESRSRKRILINPGNHLKIQEGTLGFFIASDAKEVKRAFFYCKACHDDVTDPKRIKK--CGCRR--

E3VRZ8 DDAICLAELKLGFIAQSCLAQGLSTMLANLFSMRSFIKIEEDTWQKYYLEGVSNEMYTEYLSSAFVGLSFPTVCELCFVKLKLLMIAIEYKSANRESRSRKRILINPGNHLKIQEGTLGFFIASDAKEVKRAFFYCKACHDDVTDPKRIKK--CGCRRLI

E3VRZ9 DDAICLAELKLGFIAQSCLAQGLSTMLANLFSMRPFIKIEEDTWQKYYLEGVSNEMYTEYLSSAFVGLSFPTVCELCFVKLKLLMIAIEYKSANRESR----ILINPGNHLKIQEGTLGFFIASDAKEVKRAFFYCKACHDDVTDPKRIKK--CGCRR--

E3VRZ3 DDAICLAELKLGFIAQSCLAQGLSTMLANLFSMRSFIKIEEDTWQKYYLEGVSNEMYTEYLSSAFVGLSFPTVCELCFVKLKLLMIAIEYKSANRESR----ILINPGNHLKIQEGTLGFFIASDAKEVKRAFFYCKACHDDVTDPKRIKK--CGCRR--

E3VS03 DDAICLAELKLGFIAQSCLAQGLSTMLANLFSMRSFIKIEEDTWQKYYLEGVSNEMYTEYLSSAFVGLSFPTVCELCFVKLKLLMIAIEYKSANRESR----ILINPGNHLKIQEGTLGFFIASDAKEVKRAFFYCKACHDDVTDPKRIKK--CGCRRLK

E3VS04 DDAICLAELKLGFIAQSCLAQGLSTMLANLFSMRSFIKIGEDTWQKYYLEGVSNEMYTEYPSSAFVGLSFPTVCELCFVKLKLLMIAIEYKSANRESR----ILINPGNHLKIQEGTLGFFIASDAKEVKRAFFYCKACHDDVTDPKRIKK--CGCRRL-

E3VS00 DDAICLAELKLGFIAQSCLAQGLSTMLANLFSMRSFIKIEEDTWQKYYLEGVSNEMYTEYLSSAFVGLSFPTVCELCFVKLKLLMIAIEYKSANRESR----ILINPGNHLKIQEGTLGFFIASDAKEVKRAFFYCKACHDDVTDPKRIKK--CGCRRLE

E3VS01 DDAICLAELKLGFIAQSCLAQGLSTMLANLFSMRSFIKIEEDTWQKYYLEGVSNEMYTEYLSSAFVGLSFPTVCELCFVKLKLLMIAIEYKSANRESR----ILINPGNHLKIQEGTLGFFIASDAKEVKRAFFYCKACHDDVTDPKRIKK--CGCRRLE

E3VRY4 DDAICLAELKLGFIAQSCLAQGLSTMLANLFSMRSFIKIEEDTWQKYYLEGVSNEMYTEYLSSAFVGLSFPTVCELCFVKLKLLMIAIEYKSANRESR----ILINPGNHLKIQEGTLGFFIASDAKEVKRAFFYCKACHDDVTDPKRIKK--CGCRRLI

E3VS02 DDAICLAELKLGFIAQSCLAQGLSTMLANLFSMRSFIKIEEDTWQKYYLEGVSNEMYTEYLSSAFVGLSFPTVCELCFVKLKLLMIAIEYKSANRESR----ILINPGNHLKIQEGTLGFFIASDAKEVKRAFFYCKACHDDVTDPKRIKK--CGCRR--

E3VRY5 DDAICLAELKLGFIARSCLAQGLSTMLANLFSMRSFIKIEEDTWQKYYLEGVSNEMYTEYLSSAFVGLSFPTVCELCFVKLKLLMIAIEYKSANRESR----ILINPGNHLKIQEGTLGFFIASDAKEVKRAFFYCKACHDDVTDPKRIKK--CGCRRLI

E3VRZ0 DDAICLAELKLGFIAQGCLAQGLSTMLANLFSMRSFIKIEEDTWQKYYLEGVSNEMYTEYLSSAFVGLSFPTVCELCFVKLKLLMIAIEYKSANRESR----ILINPGNHLKIQEGTLGFFIASDAKEVKRAFFYCKACHDDVTDPKRIKK--CGCRRLI

E3VRY8 DDAICLAELKLGFIAQSCLAQGLSTMLANLFSMRSFIKIEEDTWQKYYLEGVSNEMYTEYLSSAFVGLSFPTVCELCFVKLKLLMIAIEYKSANRESR----ILINPGNHLKIQEGTLGFFIASDAKEVKRAFFYCKACHDDVTDPKRIKK--CGCRRLI

E3VRZ1 DDAICLAELKLGFIAQSCLAQGLSTMLANLFSMRSFIKIEEDTWQKYYLEGVSNEMYTEYLSSAFVGLSFPTVCELCFVKLKLLMIAIEYKSANRESR----ILINPGNHLKIQEGTLGFFIASDAKEVKRAFFYCKACHDDVTDPKRIKK--CGCRRLI

C3VLD3 DDAICLAELKLGFIAQSCLAQGLSTMLANLFSMRSFIKIEEDTWQKYYLEGVSNEMYTEYLSSAFVGLSFPTVCELCFVKLKLLMIAIEYKSANRESR----ILINPGNHLKIQEGTLGFFIASDAKEVKRAFFYCKACHDDVTDPKRIKK--CGCRRLI

E3VRY9 DDAICLAELKLGFIAQSCLAQGLSTMLANLFSMRSFIKIEEDTWQKYYLEGVSNEMYTEYLSSAFVGLSFPTVCELCFVKLKLLMIAIEYKSANRESR----ILINPGNHLKIQEGTLGFFIASDAKEVKRAFFYCKACHDDVTDPKRIKK--CGCRR--

**ALIGN ***************..*****************.**** ******************** ************************************* ******************************** ... **..* .* ***

E3VRZ5 ------------------------------------------------------------EDEQPPTLSPKKKQRNGGMRNSPNTSPKLMRHDPLLIPGNDQIDNMDSNVKKYDSTGMFHWCAPKEIEKVILTRSEAAMTVLSGHVVVCIFGDVSSALIG

E3VRZ6 --------------------------------VEARARYHKDPFMHKNATPNSPHVPKPVEDEQPPTLSPKKKQRNGGMRNSPNTSPKLMRHDPLLIPGNDQIDNMDSNVKKYDSTGMFHWCAPKEIEKVILTRSEAAMTVLSGHVVVCIFGDVSSALIG

E3VRZ4 ------------------------------------------------------------EDEQPPTLSPKKKQRNGGMRNSPNTSPKLMRHDPLLIPGNDQIDNMDSNVKKYDSTGMFHWCAPKEIEKVILTRSEAAMTVLSGHVVVCIFGDVSSALIG

E3VRY6 -------------------------------------------------------------DEQPPTLSPKKKQRNGGMRNSPNTSPKLMRHDPLLIPGNDQIDNMDSNVKKYDSTGMFHWCAPKEIEKVILTRSEAAMTVLSGHVVVCIFGDVSSALIG

E3VRZ2 --------------------------------LEPAITKTHLCTRMRLPILHTCPSQLKMSSRQP---CHQKKQRNGGMRNSPNTSPKLMRHDPLLIPGNDQIDNMDSNVKKYDSTGMFHWCAPKEIEKVILTRSEAAMTVLSGHVVVCIFGDVSSALIG

E3VRY7 -------------------------------------------------------------DEQPPTLSPKKKQRNGGMRNSPNTSPKLMRHDPLLIPGNDQIDNMDSNVKKYDSTGMFHWCAPKEIEKVILTRSEAATTVLSGHVVVCIFGDVSSALIG

E3VRZ7 -PKMSIYKRMRRACCFDCGRSERDCSCMSGRVRGNVDTLERTFPLSSVSVNDCSTSFRAFEDEQPPTLSPKKKQRNGGMRNSPNTSPKLMRHDPLLIPGNDQIDNMDSNVKKYDSTGMFHWCAPKEIEKVILTRSEAAMTVLSGHVVVCIFGDVSSALIG

E3VRZ8 YSKMSIYKRMRRACCFDCGRSERDCSCMSGRVRGNVDTLERTFPLSSVSVNDCSTSFRAFEDEQPPTLSPKKKQRNGGMRNSPNTSPKLMRHDPLLIPGNDQIDNMDSNVKKYDSTGMFHWCAPKEIEKVILTRSEAAMTVLSGHVVVCIFGDVSSALIG

E3VRZ9 -PKMSIYKRMRRACCFDCGRSERDCSCMSGRVRGNVDTLERTFPLSSVSVNDCSTSFRAFEDEQPPTLSPKKKQRNGGMRNSPNTSPKLMRHDPLLIPGNDQIDNMDSNVKKYDSTGMFHWCAPKEIEKVILTRSEAAMTVLSGHVVVCIFGDVSSALIG

E3VRZ3 -----------------------------------------------------------LEDEQPPTLSPKKKQRNGGMRNSPNTSPKLMRHDPLLIPGNDQIDNMDSNVKKYDSTGMFHWCAPKEIEKVILTRSEAAMTVLSGHVVVCIFGDVSSALIG

E3VS03 --------------------------------VEARARYHKDPFMHKNATPNSPHVPKPVEDEQPPTLSPKKKQRNGGMRNSPNTSPKLMRHDPLLIPGNDQIDNMDSNVKKYDSTGMFHWCAPKEIEKVILTRSEAAMTVLSGHVVVCIFGGVSSALIG

E3VS04 ------------------------------------------------------------EDEQPPTLSPKKKQRNGGMRNSPNTSPKLMRHDPLLIPGNDQIDNMDSNVKKYDSTGMFHWCAPKEIEKVILTRSEAAMTVLSGHVVVCIFGDVSSALIG

E3VS00 -------------------------------------------------------------DEQPPTLSPKKKQRNGGMRNSPNTSPKLMRHDPLLIPGNDQIDNMDSNVKKYDSTGMFHWCAPKEIEKVILTRSEAAMTVLSGHVVVCIFGDVSSALIG

E3VS01 -------------------------------------------------------------DEQPPTLSPKKKQRNGGMRNSPNTSPKLMRHDPLLIPGNDQIDNMDSNVKKYDSTGMFHWCAPKEIEKVILTRSEAAMTVLSGHVVVCIFGDVSSALIG

E3VRY4 ----------------------------------------------------------YFEDEQPPTLSPKKKQRNGGMRNSPNTSPKLMRHDPLLIPGNDQIDNMDSNVKKYDSTGMFHWCAPKEIEKVILTRSEAAMTVLSGHVVVCIFGDVSSALIG

E3VS02 -----------------------------------------------------------LEDEQPPTLSPKKKQRNGGMRNSPNTSPKLMRHDPLLIPGNDQIDNMDSNVKKYDSTGMFHWCAPKEIEKVILTRSEAAMTVLSGHVVVCIFGDVSSALIG

E3VRY5 ----------------------------------------------------------YFEDEQPPTLSPKKKQRNGGMRNSPNTSPKLMRHDPLLIPGNDQIDNMDSNVKKYDSTGMFHWCAPKEIEKVILTRSEAAMTVLSGHVVVCIFGDVSSALIG

E3VRZ0 ----------------------------------------------------------YFEDEQPPTLSPKKKQRNGGMRNSPNTSPKLMRHDPLLIPGNDQIDNMDSNVKKYDSTGMFHWCAPKEIEKVILTRSEAAMTVLSGHVVVCIFGDVSSALIG

E3VRY8 ----------------------------------------------------------YFEDEQPPTLSPKKKQRNGGMRNSPNTSPKLMRHDPLLIPGNDQIDNMDSNVKKYDSTGMFHWCAPKEIEKVILTRSEAAMTVLSGHVVVCIFGDVSSALIG

E3VRZ1 ----------------------------------------------------------YFEDEQPPTLSPKKKQRNGGMRNSPNTSPKLMRHDPLLIPGNDQIDNMDSNVKKYDSTGMFHWCAPKEIEKVILTRSEAAMTVLSGHVVVCIFGDVSSALIG

C3VLD3 ----------------------------------------------------------YFEDEQPPTLSPKKKQRNGGMRNSPNTSPKLMRHDPLLIPGNDQIDNMDSNVKKYDSTGMFHWCAPKEIEKVILTRSEAAMTVLSGHVVVCIFGDVSSALIG

E3VRY9 -----------------------------------------------------------LEDEQPPTLSPKKKQRNGGMRNSPNTSPKLMRHDPLLIPGNDQIDNMDSNVKKYDSTGMFHWCAPKEIEKVILTRSEAAMTVLSGHVVVCIFGDVSSALIG

**ALIGN ** .******************************************************************* *************.*********

E3VRZ5 LRNLVMPLRASNFHYHELKHIVFVGSIEYLKREWETLHNFPKVSILPGTPLSRADLRAVNINLCDMCVILSANQNNIDDTSLQDKECILASLNIKSMQFDDSIGVLQANSQGFTPPGMDRSSPDNSPVHGMLRQPSITTGVNIPIIT-------------

E3VRZ6 LRNLVMPLRASNFHYHELKHIVFVGSIEYLKREWETLHNFPKVSILPGTPLSRADLRAVNINLCDMCVILSANQNNIDDTSLQDKECILASLNIKSMQFDDSIGVLQANSQGFTPPGMDRSSPDNSPVHGMLRQPSITTGVNIPIIT-------------

E3VRZ4 LRNLVMPLRASNFHYHELKHIVFVGSIEYLKREWETLHNFPKVSILPGTPLSRADLRAVNINLCDMCVILSANQNNIDDTSLQDKECILASLNIKSMQFDDSIGVLQANSQGFTPPGMDRSSPDNSPVHGMLRQPSITTGVNIPIIT-------------

E3VRY6 LRNLVMPLRASNFHYHELKHIVFVGSIEYLKREWETLHNFPKVSILPGTPLSRADLRAVNINLCDMCVILSANQNNIDDTSLQDKECILASLNIKSMQFDDSIGVLQANSQGFTPPGMDRSSPDNSPVHGMLRQPSITTGVNIPIIT-------------

E3VRZ2 LRNLVMPLRASNFHYHELKHIVFVGSIEYLKREWETLHNFPKVSILPGTPLSRADLRAVNINLCDMCVILSANQNNIDDTSLQDKECILASLNIKSMQFDDSIGVLQANSQGFTPPGMDRSSPDNSPVHGMLRQPSITTGVNIPIIT-------------

E3VRY7 LRNLVMPLRASNFHYHELKHIVFVGSIEYLKREWETLHNFPKVSILPGTPLSRADLRAVNINLCDMCVILSANQNNIDDTSLQDKECILASLNIKSMQFDDSIGVLQANSQGFTPPGMDRSSPDNSPVHGMLRQPSITTGVNIPIIT-------------

E3VRZ7 LRNLVMPLRASNFHYHELKHIVFVGSIEYLKREWETLHNFPKVSILPGTPLSRADLRAVNINLCDMCVILSANQNNIDDTSLQDKECILASLNIKSMQFDDSIGVLQANSQGFTPPGMDRSSPDNSPVHGMLRQPSITTGVNIPIIT-------------

E3VRZ8 LRNLVMPLRASNFHYHELKHIVFVGSIEYLKREWETLHNFPKVSILPGTPLSRADLRAVNINLCDMCVILSANQNNIDDTSLQDKECILASLNIKSMQFDDSIGVLQANSQGFTPPGMDRSSPDNSPVHGMLRQPSITTGVNIPIIT-------------

E3VRZ9 LRNLVMPLRASNFHYHELKHIVFVGSIEYLKREWETLHNFPKVSILPGTPLSRADLRAVNINLCDMCVILSANQNNIDDTSLQDKECILASLNIKSMQFDDSIGVLQANSQGFTPPGMDRSSPDNSPVHGMLRQPSITTGVNIPIIT-------------

E3VRZ3 LRNLVMPLRASNFHYHELKHIVFVGSIEYLKREWETLHNFPKVSILPGTPLSRADLRAVNINLCDMCVILSADQNNIDDTSLQDKECILASLNIKSMQFDDSIGVLQANSQGFTPPGMDRSSPDNSPVHGMLRQPSITTGVNILIIT-------------

E3VS03 LRNLVMPLRASNFHYHELKHIVFVGSIEYLKREWETLHNFPKVSILPGTPLSRADLRAVNINLCDMCVILSANQNNIDDTSLQDKECILASLNIKSMQFDDSIGVLQANSQGFTPPGMDRSSPDNSPVHGMLRQPSITTGVNIPIIT-------------

E3VS04 LRNLVMPLRASNFHYHELKHIVFVGSIEYLKREWETLHNFPKVSILPGTPLSRADLRAVNINLCDMCVILSANQNNIDDTSLQDKECILASLNIKSMQFDDSIGVLQANSQGFTPPGMDRSSPDNSPVHGMLRQPSITTGVNIPIIT-------------

E3VS00 LRNLVMPLRASNFHYHELKHIVFVGSIEYLKREWETLHNFPKVSILPGTPLSRADLRAVNINLCDMCVILSANQNNIDDTSLQDKECILASLNIKSMQFDDSIGVLQANSQGFTPPGMDRSSPGNSPVHGMLRQPSITTGVNIPIIT-------------

E3VS01 LRNLVMPLRASNFHYHELKHIVFVGSIEYLKREWETLHNFPKVSILPGTPLSRADLRAVNINLCDMCVILSANQNNIDDTSLQDKECILASLNIKSMQFDDSIGVLQANSQGFTPPGMDRSSPDNSPVHGMLRQPSITTGVNIPIIT-------------

E3VRY4 LRNLVMPLRASNFHYHELKHIVFVGSIEYLKREWETLHNFPKVSILPGTPLSRADLRAVNINLCDMCVILSANQNNIDDTSLQDKECILASLNIKSMQFDDSIGVLQANSQGFTPPGMDRSSPDNSPVHGMLRQPSITTGVNIPIITELAKPGKLPLVSV

E3VS02 LRNLVMPLRASNFHYHELKHIVFVGSIEYLKREWETLHNFPKVSILPGTPLSRADLRAVNINLCDMCVILSANQNNIDDTSLQDKECILASLNIKSMQFDDSIGVLQANSQGFTPPGMDRSSPDNSPVHGMLRQPSITTGVNIPIIT-------------

E3VRY5 LRNLVMPLRASNFHYHELKHIVFVGSIEYLKREWETLHNFPKVSILPGTPLSRADLRAVNINLCDMCVILSANQNNIDDTSLQDKECILASLNIKSMQFDDSIGVLQANSQGFTPPGMDRSSPDNSPVHGMLRQPSITTGVNIPIIT-------------

E3VRZ0 LRNLVMPLRASNFHYHELKHIVFVGSIEYLKREWETLHNFPKVSILPGTPLSRADLRAVNINLCDMCVILSANQNNIDDTSLQDKECILASLNIKSMQFDDSIGVLQANSQGFTPPGMDRSSPDNSPVHGMLRQPSITTGVNIPIITELAKPGKLPLVSV

E3VRY8 LRNLVMPLRASNFHYHELKHIVFVGSIEYLKREWETLHNFPKVSILPGTPLSRADLRAVNINLCDMCVILSANQNNIDDTSLQDKECILASLNIKSMQFDDSIGVLQANSQGFTPPGMDRSSPDNSPVHGMLRQPSITTGVNIPIIT-------------

E3VRZ1 LRNLAMPLRASNFHYHELKHIVFVGSIEYLKREWETLHNFPKVSILPGTPLSRADLRAVNINLCDMCVILSANQNNIDDTSLQDKECILASLNIKSMQFDDSIGVLQANSQGFTPPGMDRSSPDNSPVHGMLRQPSITTGVNIPIIT-------------

C3VLD3 LRNLVMPLRASNFHYHELKHIVFVGSIEYLKREWETLHNFPKVSILPGTPLSRADLRAVNINLCDMCVILSANQNNIDDTSLQDKECILASLNIKSMQFDDSIGVLQANSQGFTPPGMDRSSPDNSPVHGMLRQPSITTGVNIPIITELAKPGKLPLVSV

E3VRY9 LRNLVMPLRASNFHYHELKHIVFVGSIEYLKREWETLHNFPKVSILPGTPLSRADLRAVNINLCDMCVILSANQNNIDDTSLQDKECILASLNIKSMQFDDSIGVLQANSQGFTPPGMDRSSPDNSPVHGMLRQPSITTGVNIPIITELAKPGKLPLVSV

**ALIGN ****.*******************************************************************.**************************************************.******************* *****

E3VRZ5 --------------ELVNDTNVQFLDQDDDDDPDTELYLTQPFACGTAFAVSVLDSLMSATYFNDNILTLIRTLVTGGATPELEALIAEENAPRGGYSTPQTLANRDRCRVAQLALLDGPFADLGDGGCYGDLFCKALKTYNMLCFGIYRLRDAHLSTPS

E3VRZ6 --------------ELVNDTNVQFLDQDDDDDPDTELYLTQPFACGTAFAVSVLDSLMSATYFNDNILTLIRTLVTGGATPELEALIAEENALRGGYSTPQTLANRDRCRVAQLALLDGPFADLGDGGCYGDLFCKALKTYNMLCFGIYRLRDAHLSTPS

E3VRZ4 --------------ELVNDTNVQFLDQDDDDDPDTELYLTQPFACGTAFAVSVLDSLMSATYFNDNILTLIRTLVTGGATPELEALIAEENALRGGYSTPQTLANRDRCRVAQLALLDGPFADLGDGGCYGDLFCKALKTYNMLCFGIYRLRDAHLSTPS

E3VRY6 --------------ELVNDTNVQFLDQDDDDDPDTELYLTQPFACGTAFAVSVLDSLMSATYFNDNILTLIRTLVTGGATPELEALIAEENALRGGYSTPQTLANRDRCRVAQLALLDGPFADLGDGGCYGDLFCKALKTYNMLCFGIYRLRDAHLSTPS

E3VRZ2 --------------ELVNDTNVQFLDQDDDDDPDTELYLTQPFACGTAFAVSVLDSLMSATYFNDNILTLIRTLVTGGATPELEALIAEENALRGGYSTPQTLANRDRCRVAQLALLDGPFADLGDGGCYGDLFCKALKTYNMLCFGIYRLRDAHLSTPS

E3VRY7 --------------ELVNDTNVQFLDQDDDDDPDTELYLTQPFACGTAFAVSVLDSLMSATYFNDNILTLIRTLVTGGATPELEALIAEENALRGGYSTPQTLANRDRCRVAQLALLDGPFADLGDGGCYGDLFCKALKTYNMLCFGIYRLRDAHLSTPS

E3VRZ7 --------------ELVNDTNVQFLDQDDDDDPDTELYLTQPFACGTAFAVSVLDSLMSATYFNDNILTLIRTLVTGGATPELEALIAEENALRGGYSTPQTLANRDRCRVAQLALLDGPFADLGDGGCYGDLFCKALKTYNMLCFGIYRLRDAHLSTPS

E3VRZ8 --------------ELVNDTNVQFLDQDDDDDPDTELYLTQPFACGTAFAVSVLDSLMSATYFNDNILTLIRTLVTGGATPELEALIAEENALRGGYSTPQTLANRDRCRVAQLALLDGPFADLGDGGCYGDLFCKALKTYNMLCFGIYRLRDAHLSTPS

E3VRZ9 --------------ELVNDTNVQFLDQDDDDDPDTELYLTQPFACGTAFAVSVLDSLMSATYFNDNILTLIRTLVTGGATPELEALIAEENALRGGYSTPQTLANRDRCRVAQLALLDGPFADLGDGGCYGDLFCKALKTYNVLCFGIYRLRDAHLSTPS

E3VRZ3 --------------ELVNDTNVQFLDQDDDDDPDTELYLTQPFACGTAFAVSVLDSLMSATYFNDNILTLIRTLVTGGATPELEALIAEENALRGGYSTPQTLANRDRCRVAQLALLDGPFADLGDGGCYGDLFCKALKTYNMLCFGIYRLRDAHLSTPS

E3VS03 --------------ELVNDTNVQFLDQDDDDDPDTELYLTQPFACGTAFAVSVLDSLMSATYFNDNILTLIRTLVTGGATPELKALIAEENALRGGYSTPQTLANRDRCRVAQLALLDGPFADLGDGGCYGDLFCKALKTYNMLCFGIYRLRDAHLSTPS

E3VS04 --------------ELVNDTNVQFLDQDDDDDPDTELYLTQPFACGTAFAVSVLDSLMSATYFNDNILTLIRTLVTGGATPELEALIAEENALRGGYSTPQTLANRDRCRVAQLALLDGPFADLGDGGCYGDLFCKALKTYNMLCFGIYRLRDAHLSTPS

E3VS00 --------------ELVNDTNVQFLDQDDDDDPDTELYLTQPFACGTAFAVSVLDSLMSATYFNDNILTLIRTLVTGGATPELEALIAEENALRGGYSTPQTLANRDRCRVAQLALLDGPFADLGDGGCYGDLFCKALKTYNMLCFGIYRLRDAHLSTPS

E3VS01 --------------ELVNDTNVQFLDQDDDDDPDTELYLTQPFACGTAFAVSVLDSLMSATYFNDNILTLIRTLVTGGATPELEALIAEENALRGGYSTPQTLANRDRCRVAQLALLDGPFADLGDGGCYGDLFCKALKTYNMLCFGIYRLRDAHLSTPS

E3VRY4 NQEKNSGTHILMITELVNDTNVQFLDQDDDDDPDTELYLTQPFACGTAFAVSVLDSLMSATYFNDNILTLIRTLVTGGATPELEALIAEENALRGGYSTPQTLANRDRCRVAQLALLDGPFADLGDGGCYGDLFCKALKTYNMLCFGIYRLRDAHLSTPS

E3VS02 --------------ELVNDTNVQFLDQDDDDDPDTELYLTQPFACGTAFAVSVLDSLMSAAYFNDNILTLIRTLVTGGATPELEALIAEENALRGGYSTPQTLANRDRCRVAQLALLDGPFADLGDGGCYGDLFCKALKTYNMLCFGIYRLRDAHLSTPS

E3VRY5 --------------ELVNDTNVQFLDQDDDDDPDTELYLTQPFACGTAFAVSVLDSLMSATYFNDNILTLIRTLVTGGATPELEALIAEENALRGGYSTPQTLANRDRCRVAQLALLDGPFADLGDGGCYGDLFCKALKTYNMLCFGIYRLRDAHLSTPS

E3VRZ0 NQEKNSGTHILMITELVNDTNVQFLDQDDDDDPDTELYLTQPFACGTAFAVSVLDSLMSATYFNDNILTLIRTLVTGGATPELEALIAEENALRGGYSTPQTLANRDRCRVAQLALLDGPFADLGDGGCYGDLFCKALKTYNMLCFGIYRLRDAHLSTPS

E3VRY8 --------------ELVNDTNVQFLDQDDDDDPDTELYLTQPFACGTAFAVSVLDSLMSATYFNDNILTLIRTLVTGGATPELEALIAEENALRGGYSTPQTLANRDRCRVAQLALLDGPFADLGDGGCYGDLFCKALKTYNMLCFGIYRLRDAHLSTPS

E3VRZ1 --------------ELVNDTNVQFLDQDDDDDPDTELYLTQPFACGTAFAVSVLDSLMSATYFNDNILTLIRTLVTGGATPELEALIAEENALRGGYSTPQTLANRDRCRVAQLALLDGPFADLGDGGCYGDLFCKALKTYNMLCFGIYRLRDAHLSTPS

C3VLD3 NQEKNSGTHILMITELVNDTNVQFLDQDDDDDPDTELYLTQPFACGTAFAVSVLDSLMSATYFNDNILTLIRTLVTGGATPELEALIAEENALRGGYSTPQTLANRDRCRVAQLALLDGPFADLGDGGCYGDLFCKALKTYNMLCFGIYRLRDAHLSTPS

E3VRY9 NQEKNSGTHILMITELVNDTNVQFLDQDDDDDPDTELYLTQPFACGTAFAVSVLDSLMSATYFNDNILTLIRTLVTGGATPELEALIAEENALRGGYSTPQTLANRDRCRVAQLALLDGPFADLGDGGCYGDLFCKALKTYNMLCFGIYRLRDAHLSTPS

**ALIGN **********************************************.**********************.******** *************************************************.*******************

E3VRZ5 QCTKRYVITNPPYEFELVPTDLIFCLMQFDHNAGQSRASLSHSSHSSQSSSKKSSSVHSIPSTANRPNRPKSRESHDKQKYVQEERL-----------------------------------------------------

E3VRZ6 QCTKRYVITNPPYEFELVPTDLIFCLMQFDHNAGQSRASLSHSSHSSQSSSKKSSSVHSIPSTANRPNRPKSRESRDKQKYVQEERL-----------------------------------------------------

E3VRZ4 QCTKRYVITNPPYEFELVPTDLIFCLMQFDHNAGQSRASLSHSSHSSQSSSKKSSSVHSIPSTANRPNRPKSRESRDKQKYVQEERL-----------------------------------------------------

E3VRY6 QCTKRYVITNPPYEFELVPTDLIFCLMQFDHNAGQSRASLSHSSHSSQSSSKKSSSVHSIPSTANRPNRPKSRESRDKQKYVQEERL-----------------------------------------------------

E3VRZ2 QCTKRYVITNPPYEFELVPADLIFCLMQFDHNAGQSRASLSHSSHSSQSSSKKSSSVHSIPSTANRPNRPKSRESRDKQKYVQEERL-----------------------------------------------------

E3VRY7 QCTKRYVITNPPYEFELVPTDLIFCLMQFDHNAGQSRASLSHSSHSSQSSSKKSSSVHSIPSTANRPNRPKSRESRDKQKYVQEERL-----------------------------------------------------

E3VRZ7 QCTKRYVITNPPYEFELVPTDLIFCLMQFDHNAGQSRASLSHSSHSSQSSSKKSSSVHSIPSTANRPNRPKSRESRDKQNRKEMVYR-----------------------------------------------------

E3VRZ8 QCTKRYVITNPPYEFELVPTDLIFCLMQFDHNAGQSRASLSHSSHSSQSSSKKSSSVHSIPSTANRPNRPKSRESRDKQNRKEMVYR-----------------------------------------------------

E3VRZ9 QCTKRYVITNPPYEFELVPTDLIFCLMQFDHNAGQSRASLSHSSHSSQSSSKKSSSVHSIPSTANRPNRPKSRESRDKQKYVQEERL-----------------------------------------------------

E3VRZ3 QCTKRYVITNPPYEFELVPTDLIFCLMQFDHNAGQSRASLSHSSHSSQSSSKKSSSVHSIPSTANRPNRPKSRESRDKQSRKEMVYR-----------------------------------------------------

E3VS03 QCTKRYVITNPPYEFELVPTDLIFCLMQFDHNAGQSRASLSHSSHSSQSSSKKSSSVHSIPSTANRPNRPKSRESRDKQNRKEMVYR-----------------------------------------------------

E3VS04 QCTKRYVITNPPYEFELVPTDLIFCLMQFDHNAGQSRASLSHSSHSSQSSSKKSSSVHSIPSTANRPNRPKSRESRDKQNRKEMVYR-----------------------------------------------------

E3VS00 QCTKRYVITNPPYEFELVPTDLIFCLMQFDHNAGQSRASLSHSSHSSQSSSKKSSSVHSIPSTANRPNRPKSRESRDKQKKEVAYR------------------------------------------------------

E3VS01 QCTKRYVITNPPYEFELVPTDLIFCLMQFDHNAGQSRASLSHSSHSSQSSSKKSSSVHSIPSTANRPNRPKSRESRDKQKKEMVYR------------------------------------------------------

E3VRY4 QCTKRYVITNPPYEFELVPTDLIFCLMQFDHNAGQSRASLSHSSHSSQSSSKKSSSVHSIPSTANRPNRPKSRESRDKQKKEMVYR------------------------------------------------------

E3VS02 QCTKRYVITNPPYEFELVPTDLIFCLMQFDHNAGQSRASLSHSSHSSQSSSKKSSSVHSIPSTANRPNRPKSRESRDKQKKEMVYR------------------------------------------------------

E3VRY5 QCTKRYVITNPPYEFELVPTDLIFCLMQFDHNAGQSRASLSHSSHSSQSSSKKSSSVHSIPSTANRPNRPKSRESRDKQNATRMTRMGQAEKKWFTDEPDNAYPRNIQIKPMSTHMANQINQYKSTSSLIPPIREVEDEC

E3VRZ0 QCTKRYVITNPPYEFELVPTDLIFCLMQFDHNAGQSRASLSHSSHSSQSSSKKSSSVHSIPSTANRPNRPKSRESRDKQNATRMTRMGQAEKKWFTDEPDNAYPRNIQIKPMSTHMANQINQYKSTSSLIPPIREVEDEC

E3VRY8 QCTKRYVITNPPYEFELVPTDLIFCLMQFDHNAGQSRASLSHSSHSSQSSSKKSSSVHSIPSTANRPNRPKSRESRDKQNATRMTRMGQAEKKWFTDEPDNAYPRNIQIKPMSTHMANQINQYKSTSSLIPPIREVEDEC

E3VRZ1 QCTKRYVITNPPYEFELVPTDLIFCLMQFDHNAGQSRASLSHSSHSSQSSSKKSSSVHSIPSTANRPNRPKSRESRDKQNATRMTRMGQ-EKKWFTDEPDNAYPRNIQIKPMSTHMANQINQYKSTSSLIPPIREVEDEC

C3VLD3 QCTKRYVITNPPYEFELVPTDLIFCLMQFDHNAGQSRASLSHSSHSSQSSSKKSSSVHSIPSTANRPNRPKSRESRDKQNATRMTRMGQ-EKKWFTDEPDNAYPRNIQIKPMSTHMANQINQYKSTSSLIPPIREVEDEC

E3VRY9 QCTKRYVITNPPYEFELVPTDLIFCLMQFDHNAGQSRASLSHSSHSSQSSSKKSSSVHSIPSTANRPNRPKSRESRDKQNATRMTRMGQ-EKKWFTDEPDNAYPRNIQIKPMSTHMANQINQYKSTSSLIPPIREVEDEC

**ALIGN *******************.*******************************************************.***.**

# Aligned disorder predictions for BK channel variants

E3VRZ5 1111111111111111111111111111111111

E3VRZ6 111111111111111111111111111111111

E3VRZ4 1111111111111111111111111111111111

E3VRY6 1111111111111111111111111111111111

E3VRZ2 111111111111111111111111111111111

E3VRY7 1111111111111111111111111111111111

E3VRZ7 11111111111111111111111111111111111

E3VRZ8 11111111111111111111111111111111

E3VRZ9 11111111111111111111111111111111111

E3VRZ3 1111111111111111111111111111111111

E3VS03 111111111111111111111111111111111

E3VS04 1111111111111111111111111111111111

E3VS00 1111111111111111111111111111111111

E3VS01 1111111111111111111111111111111111

E3VRY4 111111111111111111111111111111111

E3VS02 1111111111111111111111111111111111

E3VRY5 ------------------------------------------------------

E3VRZ0 11111111111111111111111111111111

E3VRY8 11111111111111111111111111111111

E3VRZ1 111111111111111111111111111111111

C3VLD3 111111111111111111111111111111111

E3VRY9 1111111111111111111111111111111111

**ALIGN *********.*****************************.********************* ***********************************************

E3VRZ5

E3VRZ6

E3VRZ4

E3VRY6

E3VRZ2

E3VRY7

E3VRZ7

E3VRZ8

E3VRZ9

E3VRZ3

E3VS03

E3VS04

E3VS00

E3VS01

E3VRY4

E3VS02

E3VRY5

E3VRZ0

E3VRY8

E3VRZ1

C3VLD3

E3VRY9

**ALIGN ***********************************.*******************************************.**************************************** ***********************.*********** *****

E3VRZ5

E3VRZ6

E3VRZ4

E3VRY6

E3VRZ2

E3VRY7

E3VRZ7

E3VRZ8

E3VRZ9

E3VRZ3

E3VS03

E3VS04

E3VS00

E3VS01

E3VRY4

E3VS02

E3VRY5

E3VRZ0

E3VRY8

E3VRZ1

C3VLD3

E3VRY9

**ALIGN ** ** **.*.**... .**************************************.***********************.************.******************************************************************

E3VRZ5 ---- 111111111--111111-

E3VRZ6 ---- 111111--1111111

E3VRZ4 ---- 111111111--111111-

E3VRY6 ---- 111111111--1111111

E3VRZ2 1111

E3VRY7 ---- 111111111--1111111

E3VRZ7 111111--11 --

E3VRZ8 -- 111

E3VRZ9 ---- -- --

E3VRZ3 ---- 111111111--11111--

E3VS03 ---- 11111--1111111

E3VS04 ---- 1111111111--111111-

E3VS00 ---- 111111111--1111111

E3VS01 ---- 111111111--1111111

E3VRY4 ---- 11--1111111

E3VS02 ---- 111111111--11111--

E3VRY5 ---- 1111--1111111

E3VRZ0 ---- -- 1111

E3VRY8 ---- -- 11

E3VRZ1 ---- -- 1111

C3VLD3 ---- -- 11111

E3VRY9 ---- 111111111--11111--

**ALIGN ***************..*****************.**** ******************** ************************************* ******************************** ... **..* .* ***

E3VRZ5 ------------------------------------------------------------1111111111111111111111111111111111111111111111111111111111111

E3VRZ6 --------------------------------11111111111111111111111111111111111111111111111111111111111111111111111111111111111111

E3VRZ4 ------------------------------------------------------------1111111111111111111111111111111111111111111111111111111111111

E3VRY6 -------------------------------------------------------------111111111111111111111111111111111111111111111111111111111111

E3VRZ2 --------------------------------111111111111111111111111111111111---111111111111111111111111111111111111111111111111111

E3VRY7 -------------------------------------------------------------1111111111111111111111111111111111111111111111111111111111111

E3VRZ7 -1111111111111111111111111111111111111111111111111111111111111111111111111111111111111111111111111111111111111111111

E3VRZ8 111111111111111111111111111111111111111111111111111111111111111111111111111111111111111111111111111111111111111111

E3VRZ9 -11111111111111111111111111111111111111111111111111111111111111111111111111111111111111111111111111111111111111111

E3VRZ3 -----------------------------------------------------------111111111111111111111111111111111111111111111111111111111111111

E3VS03 --------------------------------111111111111111111111111111111111111111111111111111111111111111111111111111111111111111

E3VS04 ------------------------------------------------------------1111111111111111111111111111111111111111111111111111111111111

E3VS00 -------------------------------------------------------------1111111111111111111111111111111111111111111111111111111111111

E3VS01 -------------------------------------------------------------111111111111111111111111111111111111111111111111111111111111

E3VRY4 ----------------------------------------------------------111111111111111111111111111111111111111111111111111111111111111

E3VS02 -----------------------------------------------------------11111111111111111111111111111111111111111111111111111111111111

E3VRY5 ----------------------------------------------------------1111111111111111111111111111111111111111111111111111111111111

E3VRZ0 ----------------------------------------------------------111111111111111111111111111111111111111111111111111111111111111

E3VRY8 ----------------------------------------------------------111111111111111111111111111111111111111111111111111111111111

E3VRZ1 ----------------------------------------------------------1111111111111111111111111111111111111111111111111111111111111

C3VLD3 ----------------------------------------------------------111111111111111111111111111111111111111111111111111111111111111

E3VRY9 -----------------------------------------------------------11111111111111111111111111111111111111111111111111111111111111111

**ALIGN ** .******************************************************************* *************.*********

E3VRZ5 111111111111111111 -------------

E3VRZ6 111111111111111111111111 -------------

E3VRZ4 1111111111111111111111111 -------------

E3VRY6 11111111111111111 -------------

E3VRZ2 11111111111111111111111111 -------------

E3VRY7 11111111111111111111 -------------

E3VRZ7 1111111111111111111111111 -------------

E3VRZ8 11111111111111111111111111111 -------------

E3VRZ9 11111111111111111111111111 -------------

E3VRZ3 111111111111111111111 -------------

E3VS03 111111111111111111111 -------------

E3VS04 111111111111111111 -------------

E3VS00 111111111111111111 -------------

E3VS01 111111111111111111 -------------

E3VRY4 111111111111111111111111

E3VS02 111111111111111111 -------------

E3VRY5 1111111111111111111 -------------

E3VRZ0 1111111111111111111

E3VRY8 1111111111111111111 -------------

E3VRZ1 11111111111111111111111111 -------------

C3VLD3 1111111111111111111

E3VRY9 1111111111111111111111

**ALIGN ****.*******************************************************************.**************************************************.******************* *****

E3VRZ5 -------------- 1111111111

E3VRZ6 -------------- 111111

E3VRZ4 --------------

E3VRY6 --------------

E3VRZ2 --------------

E3VRY7 --------------

E3VRZ7 -------------- 111

E3VRZ8 -------------- 1111

E3VRZ9 -------------- 111

E3VRZ3 --------------

E3VS03 -------------- 11111111

E3VS04 --------------

E3VS00 -------------- 111

E3VS01 -------------- 1111

E3VRY4

E3VS02 -------------- 111

E3VRY5 --------------

E3VRZ0 111

E3VRY8 -------------- 111

E3VRZ1 --------------

C3VLD3

E3VRY9 1111

**ALIGN **********************************************.**********************.******** *************************************************.*******************

E3VRZ5 111111111111111111111111111111111111111111111111111111111-----------------------------------------------------

E3VRZ6 11111111111111111111111111111111111111111111111111111111111-----------------------------------------------------

E3VRZ4 111111111111111111111111111111111111111111111111111111111-----------------------------------------------------

E3VRY6 1111111111111111111111111111111111111111111111111111111111-----------------------------------------------------

E3VRZ2 111111111111111111111111111111111111111111111111111111111-----------------------------------------------------

E3VRY7 1111111111111111111111111111111111111111111111111111111111-----------------------------------------------------

E3VRZ7 11111111111111111111111111111111111111111111111111111111111-----------------------------------------------------

E3VRZ8 11111111111111111111111111111111111111111111111111111111111-----------------------------------------------------

E3VRZ9 11111111111111111111111111111111111111111111111111111111111-----------------------------------------------------

E3VRZ3 11111111111111111111111111111111111111111111111111111111111-----------------------------------------------------

E3VS03 1111111111111111111111111111111111111111111111111111111111-----------------------------------------------------

E3VS04 111111111111111111111111111111111111111111111111111111111-----------------------------------------------------

E3VS00 11111111111111111111111111111111111111111111111111111111------------------------------------------------------

E3VS01 11111111111111111111111111111111111111111111111111111111------------------------------------------------------

E3VRY4 1111111111111111111111111111111111111111111111111111111------------------------------------------------------

E3VS02 111111111111111111111111111111111111111111111111111111------------------------------------------------------

E3VRY5 1111111111111111111111111111111111111111111111111111111111111111111111111111111111111111111111111111111111111111

E3VRZ0 1111111111111111111111111111111111111111111111111111111111111111111111111111111111111111111111111111111111111111

E3VRY8 1111111111111111111111111111111111111111111111111111111111111111111111111111111111111111111111111111111111111111

E3VRZ1 111111111111111111111111111111111111111111111111111111111111-11111111111111111111111111111111111111111111111111

C3VLD3 1111111111111111111111111111111111111111111111111111111111111-11111111111111111111111111111111111111111111111111

E3VRY9 1111111111111111111111111111111111111111111111111111111111111-11111111111111111111111111111111111111111111111111

**ALIGN *******************.*******************************************************.***.**

# Aligned MoRF predictions for BK channel variants

E3VRZ5 11 1

E3VRZ6 1

E3VRZ4 11 1

E3VRY6 11

E3VRZ2

E3VRY7 11 1

E3VRZ7 1 1

E3VRZ8

E3VRZ9

E3VRZ3 1 1

E3VS03

E3VS04 1

E3VS00 11 1

E3VS01 1

E3VRY4 1

E3VS02 1

E3VRY5 1 ------------------------------------------------------

E3VRZ0 1

E3VRY8 1

E3VRZ1 1

C3VLD3

E3VRY9 1

**ALIGN *********.*****************************.********************* ***********************************************

E3VRZ5 11

E3VRZ6 11

E3VRZ4 11

E3VRY6 11

E3VRZ2 11

E3VRY7 11

E3VRZ7 11

E3VRZ8 11

E3VRZ9 11

E3VRZ3 11

E3VS03 11

E3VS04 11

E3VS00 11

E3VS01 11

E3VRY4 11

E3VS02 11

E3VRY5 11

E3VRZ0 11

E3VRY8 11

E3VRZ1 11

C3VLD3 11

E3VRY9 11

**ALIGN ***********************************.*******************************************.**************************************** ***********************.*********** *****

E3VRZ5

E3VRZ6

E3VRZ4 111

E3VRY6

E3VRZ2

E3VRY7

E3VRZ7 1

E3VRZ8

E3VRZ9

E3VRZ3

E3VS03

E3VS04

E3VS00

E3VS01 11

E3VRY4

E3VS02

E3VRY5 11

E3VRZ0

E3VRY8

E3VRZ1 11

C3VLD3

E3VRY9

**ALIGN ** ** **.*.**... .**************************************.***********************.************.******************************************************************

E3VRZ5 1 ---- 11--11 -

E3VRZ6 1 ---- --

E3VRZ4 1 ---- 1 11--111 -

E3VRY6 1 ---- 1--11

E3VRZ2

E3VRY7 1 ---- 1 11--11

E3VRZ7 -- --

E3VRZ8 --

E3VRZ9 1 ---- -- --

E3VRZ3 1 ---- 1--1 --

E3VS03 1 ---- 1 --

E3VS04 1 ---- 1--1 -

E3VS00 1 ---- 1--11

E3VS01 1 ---- 1 11--111

E3VRY4 1 ---- 1 -- 111111

E3VS02 1 ---- 1--11 --

E3VRY5 1 ---- 1 -- 111111

E3VRZ0 1 ---- 1 -- 11111

E3VRY8 1 ---- 1 -- 11111

E3VRZ1 1 ---- -- 11111

C3VLD3 1 ---- 1 -- 111111

E3VRY9 1 ---- 111--11 --

**ALIGN ***************..*****************.**** ******************** ************************************* ******************************** ... **..* .* ***

E3VRZ5 ------------------------------------------------------------ 11111 1 1

E3VRZ6 -------------------------------- 111111111 11

E3VRZ4 ------------------------------------------------------------ 11111 1111

E3VRY6 ------------------------------------------------------------- 11111

E3VRZ2 -------------------------------- 1111 --- 1 1111 11 1

E3VRY7 ------------------------------------------------------------- 11111 11111 1111

E3VRZ7 - 1111 1 11111111 11111111 1111

E3VRZ8 1111111 1 111111111 1111 1

E3VRZ9 - 111 1 11 111111111 11

E3VRZ3 ----------------------------------------------------------- 11111 11 1

E3VS03 -------------------------------- 111111111 1 111

E3VS04 ------------------------------------------------------------ 11111 111

E3VS00 ------------------------------------------------------------- 11111 11

E3VS01 ------------------------------------------------------------- 11111

E3VRY4 ----------------------------------------------------------11111 1111111 11111 11

E3VS02 ----------------------------------------------------------- 11111

E3VRY5 ----------------------------------------------------------11111 11111 11111

E3VRZ0 ----------------------------------------------------------111111 11111 1111 1

E3VRY8 ----------------------------------------------------------1111111 11111 1111

E3VRZ1 ----------------------------------------------------------111111 11111 11111

C3VLD3 ----------------------------------------------------------11111 111111 1111 1

E3VRY9 ----------------------------------------------------------- 1111 11111 1

**ALIGN ** .******************************************************************* *************.*********

E3VRZ5 1 1 1-------------

E3VRZ6 1 -------------

E3VRZ4 1 -------------

E3VRY6 1 1 1-------------

E3VRZ2 1 -------------

E3VRY7 1 1 1-------------

E3VRZ7 1 -------------

E3VRZ8 1 -------------

E3VRZ9 1 -------------

E3VRZ3 1 111 1-------------

E3VS03 1 -------------

E3VS04 1 1 1-------------

E3VS00 1 1-------------

E3VS01 1 1 -------------

E3VRY4

E3VS02 1 1 -------------

E3VRY5 1 1 -------------

E3VRZ0 1 1

E3VRY8 1 1 -------------

E3VRZ1 1 -------------

C3VLD3 1 1

E3VRY9 1 11

**ALIGN ****.*******************************************************************.**************************************************.******************* *****

E3VRZ5 -------------- 1111 1 1

E3VRZ6 -------------- 11

E3VRZ4 -------------- 1 11 1 1 111

E3VRY6 -------------- 1 111 1 1

E3VRZ2 -------------- 11 1 1 1

E3VRY7 -------------- 1 1

E3VRZ7 -------------- 11 1

E3VRZ8 -------------- 111 1

E3VRZ9 -------------- 11 1

E3VRZ3 -------------- 1 1 1

E3VS03 -------------- 1111

E3VS04 -------------- 1111

E3VS00 -------------- 111 1 111

E3VS01 -------------- 111 1 1

E3VRY4 1 1

E3VS02 -------------- 111 1

E3VRY5 -------------- 111

E3VRZ0 1

E3VRY8 -------------- 11

E3VRZ1 -------------- 1111

C3VLD3 1 1

E3VRY9 1

**ALIGN **********************************************.**********************.******** *************************************************.*******************

E3VRZ5 1 1 111111111-----------------------------------------------------

E3VRZ6 1111 111111111-----------------------------------------------------

E3VRZ4 111111111-----------------------------------------------------

E3VRY6 1 1 1111 111111111-----------------------------------------------------

E3VRZ2 111111111-----------------------------------------------------

E3VRY7 1 1111 111111111-----------------------------------------------------

E3VRZ7 1 1 1 1 111111111-----------------------------------------------------

E3VRZ8 111111111-----------------------------------------------------

E3VRZ9 1 111111111-----------------------------------------------------

E3VRZ3 1 111111111-----------------------------------------------------

E3VS03 111111111-----------------------------------------------------

E3VS04 111111111-----------------------------------------------------

E3VS00 111111111------------------------------------------------------

E3VS01 111111111------------------------------------------------------

E3VRY4 111111111------------------------------------------------------

E3VS02 111111111------------------------------------------------------

E3VRY5 1 1 111 111111 111 11 11111111

E3VRZ0 1 11 111 11111 1111 1 11111

E3VRY8 1 11 111 11 1 1 11 1 11111

E3VRZ1 1 1 111 11-1111 111 11 11111111

C3VLD3 1 11 111 11-1111 111 11 11111111

E3VRY9 1 11 111 11-1111 111 11 11111111

**ALIGN *******************.*******************************************************.***.**

# Aligned globular domains predictions for BK channel variants

E3VRZ5 11111111111111111111111 11111111111111111111 1111111111111

E3VRZ6 11111111111111111111111 11111111111111111111 1111111111111

E3VRZ4 11111111111111111111111 11111111111111111111 1111111111111

E3VRY6 11111111111111111111111 11111111111111111111 1111111111111

E3VRZ2 11111111111111111111111 11111111111111111111 1111111111111

E3VRY7 11111111111111111111111 11111111111111111111 1111111111111

E3VRZ7 11111111111111111111111 11111111111111111111 1111111111111

E3VRZ8 11111111111111111111111 11111111111111111111 1111111111111

E3VRZ9 11111111111111111111111 11111111111111111111 1111111111111

E3VRZ3 11111111111111111111111 11111111111111111111 1111111111111

E3VS03 11111111111111111111111 11111111111111111111 1111111111111

E3VS04 11111111111111111111111 11111111111111111111 1111111111111

E3VS00 11111111111111111111111 11111111111111111111 1111111111111

E3VS01 11111111111111111111111 11111111111111111111 1111111111111

E3VRY4 11111111111111111111111 11111111111111111111 1111111111111

E3VS02 11111111111111111111111 11111111111111111111 1111111111111

E3VRY5 11111111111111111111111 ------------------------------------------------------111111111111111111 1111111111111

E3VRZ0 11111111111111111111111 11111111111111111111 1111111111111

E3VRY8 11111111111111111111111 11111111111111111111 1111111111111

E3VRZ1 11111111111111111111111 11111111111111111111 1111111111111

C3VLD3 11111111111111111111111 11111111111111111111 1111111111111

E3VRY9 11111111111111111111111 11111111111111111111 1111111111111

**ALIGN *********.*****************************.********************* ***********************************************

E3VRZ5 1111111111 11111111111111111111111111111111111111111111111111111111111111111111111111111111111111

E3VRZ6 1111111111 11111111111111111111111111111111111111111111111111111111111111111111111111111111111111

E3VRZ4 1111111111 11111111111111111111111111111111111111111111111111111111111111111111111111111111111111

E3VRY6 1111111111 11111111111111111111111111111111111111111111111111111111111111111111111111111111111111

E3VRZ2 1111111111 11111111111111111111111111111111111111111111111111111111111111111111111111111111111111

E3VRY7 1111111111 11111111111111111111111111111111111111111111111111111111111111111111111111111111111111

E3VRZ7 1111111111 11111111111111111111111111111111111111111111111111111111111111111111111111111111111111

E3VRZ8 1111111111 11111111111111111111111111111111111111111111111111111111111111111111111111111111111111

E3VRZ9 1111111111 11111111111111111111111111111111111111111111111111111111111111111111111111111111111111

E3VRZ3 1111111111 11111111111111111111111111111111111111111111111111111111111111111111111111111111111111

E3VS03 1111111111 11111111111111111111111111111111111111111111111111111111111111111111111111111111111111

E3VS04 1111111111 11111111111111111111111111111111111111111111111111111111111111111111111111111111111111

E3VS00 1111111111 11111111111111111111111111111111111111111111111111111111111111111111111111111111111111

E3VS01 1111111111 11111111111111111111111111111111111111111111111111111111111111111111111111111111111111

E3VRY4 1111111111 11111111111111111111111111111111111111111111111111111111111111111111111111111111111111

E3VS02 1111111111 11111111111111111111111111111111111111111111111111111111111111111111111111111111111111

E3VRY5 1111111111 11111111111111111111111111111111111111111111111111111111111111111111111111111111111111

E3VRZ0 1111111111 11111111111111111111111111111111111111111111111111111111111111111111111111111111111111

E3VRY8 1111111111 11111111111111111111111111111111111111111111111111111111111111111111111111111111111111

E3VRZ1 1111111111 11111111111111111111111111111111111111111111111111111111111111111111111111111111111111

C3VLD3 1111111111 11111111111111111111111111111111111111111111111111111111111111111111111111111111111111

E3VRY9 1111111111 11111111111111111111111111111111111111111111111111111111111111111111111111111111111111

**ALIGN ***********************************.*******************************************.**************************************** ***********************.*********** *****

E3VRZ5 11111111 1111111111111111111111111111111111111111111111111111111111111111111111111111111111111111111111111111111111111111

E3VRZ6 11111111 1111111111111111111111111111111111111111111111111111111111111111111111111111111111111111111111111111111111111111

E3VRZ4 11111111 1111111111111111111111111111111111111111111111111111111111111111111111111111111111111111111111111111111111111111

E3VRY6 11111111 1111111111111111111111111111111111111111111111111111111111111111111111111111111111111111111111111111111111111111

E3VRZ2 11111111 1111111111111111111111111111111111111111111111111111111111111111111111111111111111111111111111111111111111111111

E3VRY7 11111111 1111111111111111111111111111111111111111111111111111111111111111111111111111111111111111111111111111111111111111

E3VRZ7 11111111 1111111111111111111111111111111111111111111111111111111111111111111111111111111111111111111111111111111111111111

E3VRZ8 11111111 1111111111111111111111111111111111111111111111111111111111111111111111111111111111111111111111111111111111111111

E3VRZ9 11111111 111111111111111111111111111111111111111111111111111111111111111111111111111111111111111111111111111111111

E3VRZ3 11111111 1111111111111111111111111111111111111111111111111111111111111111111111111111111111111111111111111111111111111111

E3VS03 11111111 1111111111111111111111111111111111111111111111111111111111111111111111111111111111111111111111111111111111111111

E3VS04 11111111 1111111111111111111111111111111111111111111111111111111111111111111111111111111111111111111111111111111111111111

E3VS00 11111111 1111111111111111111111111111111111111111111111111111111111111111111111111111111111111111111111111111111111111111

E3VS01 11111111 1111111111111111111111111111111111111111111111111111111111111111111111111111111111111111111111111111111111111111

E3VRY4 11111111

E3VS02 11111111 1111111111111111111111111111111111111111111111111111111111111111111111111111111111111111111111111111111111111111

E3VRY5 11111111 1111111111111111111111111111111111111111111111111111111111111111111111111111111111111111111111111111111111111111

E3VRZ0 11111111

E3VRY8 11111111 1111111111111111111111111111111111111111111111111111111111111111111111111111111111111111111111111111111111111111

E3VRZ1 11111111 1111111111111111111111111111111111111111111111111111111111111111111111111111111111111111111111111111111111111111

C3VLD3 11111111 1111111111111111111111111111111111111111111111111111111111111111111111111111111111111111111111111111111111111111

E3VRY9 11111111 1111111111111111111111111111111111111111111111111111111111111111111111111111111111111111111111111111111111111111

**ALIGN ** ** **.*.**... .**************************************.***********************.************.******************************************************************

E3VRZ5 ---- -- -

E3VRZ6 ---- --

E3VRZ4 ---- -- -

E3VRY6 ---- --

E3VRZ2

E3VRY7 ---- --

E3VRZ7 -- --

E3VRZ8 --

E3VRZ9 ---- -- --

E3VRZ3 ---- -- --

E3VS03 ---- --

E3VS04 ---- -- -

E3VS00 ---- --

E3VS01 ---- --

E3VRY4 ---- --

E3VS02 ---- -- --

E3VRY5 ---- --

E3VRZ0 ---- --

E3VRY8 ---- --

E3VRZ1 ---- --

C3VLD3 ---- --

E3VRY9 ---- -- --

**ALIGN ***************..*****************.**** ******************** ************************************* ******************************** ... **..* .* ***

E3VRZ5 ------------------------------------------------------------

E3VRZ6 --------------------------------

E3VRZ4 ------------------------------------------------------------

E3VRY6 -------------------------------------------------------------

E3VRZ2 -------------------------------- ---

E3VRY7 -------------------------------------------------------------

E3VRZ7 -

E3VRZ8

E3VRZ9 -

E3VRZ3 -----------------------------------------------------------

E3VS03 --------------------------------

E3VS04 ------------------------------------------------------------

E3VS00 -------------------------------------------------------------

E3VS01 -------------------------------------------------------------

E3VRY4 ----------------------------------------------------------

E3VS02 -----------------------------------------------------------

E3VRY5 ----------------------------------------------------------

E3VRZ0 ----------------------------------------------------------

E3VRY8 ----------------------------------------------------------

E3VRZ1 ----------------------------------------------------------

C3VLD3 ----------------------------------------------------------

E3VRY9 -----------------------------------------------------------

**ALIGN ** .******************************************************************* *************.*********

E3VRZ5 -------------

E3VRZ6 -------------

E3VRZ4 -------------

E3VRY6 -------------

E3VRZ2 -------------

E3VRY7 -------------

E3VRZ7 -------------

E3VRZ8 -------------

E3VRZ9 -------------

E3VRZ3 -------------

E3VS03 -------------

E3VS04 -------------

E3VS00 -------------

E3VS01 -------------

E3VRY4

E3VS02 -------------

E3VRY5 -------------

E3VRZ0

E3VRY8 -------------

E3VRZ1 -------------

C3VLD3

E3VRY9

**ALIGN ****.*******************************************************************.**************************************************.******************* *****

E3VRZ5 --------------

E3VRZ6 --------------

E3VRZ4 --------------

E3VRY6 --------------

E3VRZ2 --------------

E3VRY7 --------------

E3VRZ7 --------------

E3VRZ8 --------------

E3VRZ9 --------------

E3VRZ3 --------------

E3VS03 --------------

E3VS04 --------------

E3VS00 --------------

E3VS01 --------------

E3VRY4

E3VS02 --------------

E3VRY5 --------------

E3VRZ0

E3VRY8 --------------

E3VRZ1 --------------

C3VLD3

E3VRY9

**ALIGN **********************************************.**********************.******** *************************************************.*******************

E3VRZ5 -----------------------------------------------------

E3VRZ6 -----------------------------------------------------

E3VRZ4 -----------------------------------------------------

E3VRY6 -----------------------------------------------------

E3VRZ2 -----------------------------------------------------

E3VRY7 -----------------------------------------------------

E3VRZ7 -----------------------------------------------------

E3VRZ8 -----------------------------------------------------

E3VRZ9 -----------------------------------------------------

E3VRZ3 -----------------------------------------------------

E3VS03 -----------------------------------------------------

E3VS04 -----------------------------------------------------

E3VS00 ------------------------------------------------------

E3VS01 ------------------------------------------------------

E3VRY4 ------------------------------------------------------

E3VS02 ------------------------------------------------------

E3VRY5

E3VRZ0

E3VRY8

E3VRZ1 -

C3VLD3 -

E3VRY9 -

**ALIGN *******************.*******************************************************.***.**

# Aligned cleavage sites (CLV) annotated using ELMs for BK channel variants

E3VRZ5 11111 11111

E3VRZ6 11111 11111

E3VRZ4 11111 11111

E3VRY6 11111 11111

E3VRZ2 11111 11111

E3VRY7 11111 11111

E3VRZ7 11111

E3VRZ8 11111

E3VRZ9 11111

E3VRZ3 11111 11111

E3VS03 11111

E3VS04 11111

E3VS00 11111

E3VS01 11111

E3VRY4 11111 11111

E3VS02 11111

E3VRY5 11111 ------------------------------------------------------

E3VRZ0 11111 11111

E3VRY8 11111 11111

E3VRZ1 11111 11111

C3VLD3 11111 11111

E3VRY9 11111 11111

**ALIGN *********.*****************************.********************* ***********************************************

E3VRZ5

E3VRZ6

E3VRZ4

E3VRY6

E3VRZ2

E3VRY7

E3VRZ7

E3VRZ8

E3VRZ9

E3VRZ3

E3VS03

E3VS04

E3VS00

E3VS01

E3VRY4

E3VS02

E3VRY5

E3VRZ0

E3VRY8

E3VRZ1

C3VLD3

E3VRY9

**ALIGN ***********************************.*******************************************.**************************************** ***********************.*********** *****

E3VRZ5 111

E3VRZ6 111

E3VRZ4 111

E3VRY6

E3VRZ2

E3VRY7

E3VRZ7

E3VRZ8

E3VRZ9 11111

E3VRZ3

E3VS03

E3VS04

E3VS00

E3VS01

E3VRY4 11111 11111 11111 11111

E3VS02

E3VRY5

E3VRZ0 11111 11111 11111 11111

E3VRY8

E3VRZ1

C3VLD3

E3VRY9

**ALIGN ** ** **.*.**... .**************************************.***********************.************.******************************************************************

E3VRZ5 ---- 111111111 11111-- 111-

E3VRZ6 ---- 111111111 11111-- 1111

E3VRZ4 ---- 111111111 11111-- 111-

E3VRY6 ---- 111111111 11111--

E3VRZ2 11111 111111111 11111

E3VRY7 ---- 111111111 11111--

E3VRZ7 11111 111111111 11111-- --

E3VRZ8 11111 111111111 11111--

E3VRZ9 ---- 111111111 11111-- --

E3VRZ3 ---- 111111111 11111-- --

E3VS03 ---- 111111111 11111-- 1111

E3VS04 ---- 111111111 11111-- -

E3VS00 ---- 111111111 11111--

E3VS01 ---- 111111111 11111--

E3VRY4 ---- 111111111 11111--

E3VS02 ---- 111111111 11111-- --

E3VRY5 ---- 111111111 11111--

E3VRZ0 ---- 111111111 11111--

E3VRY8 ---- 111111111 11111--

E3VRZ1 ---- 111111111 11111--

C3VLD3 ---- 111111111 11111--

E3VRY9 ---- 111111111 11111-- --

**ALIGN ***************..*****************.**** ******************** ************************************* ******************************** ... **..* .* ***

E3VRZ5 ------------------------------------------------------------ 11111111 11111

E3VRZ6 --------------------------------1 11111 11111111 11111

E3VRZ4 ------------------------------------------------------------ 11111111 11111

E3VRY6 ------------------------------------------------------------- 11111111 11111

E3VRZ2 -------------------------------- --- 11111111 11111

E3VRY7 ------------------------------------------------------------- 11111111 11111

E3VRZ7 - 11111

E3VRZ8 11111

E3VRZ9 - 11111

E3VRZ3 ----------------------------------------------------------- 11111111 11111

E3VS03 --------------------------------1 11111 11111

E3VS04 ------------------------------------------------------------ 11111

E3VS00 ------------------------------------------------------------- 11111

E3VS01 ------------------------------------------------------------- 11111

E3VRY4 ---------------------------------------------------------- 11111111 11111

E3VS02 ----------------------------------------------------------- 11111

E3VRY5 ---------------------------------------------------------- 11111111 11111

E3VRZ0 ---------------------------------------------------------- 11111111 11111

E3VRY8 ---------------------------------------------------------- 11111111 11111

E3VRZ1 ---------------------------------------------------------- 11111111 11111

C3VLD3 ---------------------------------------------------------- 11111111 11111

E3VRY9 ----------------------------------------------------------- 11111111 11111

**ALIGN ** .******************************************************************* *************.*********

E3VRZ5 111 11111 -------------

E3VRZ6 111 11111 -------------

E3VRZ4 111 11111 -------------

E3VRY6 11111 -------------

E3VRZ2 11111 -------------

E3VRY7 11111 -------------

E3VRZ7 -------------

E3VRZ8 -------------

E3VRZ9 -------------

E3VRZ3 11111 -------------

E3VS03 -------------

E3VS04 -------------

E3VS00 -------------

E3VS01 -------------

E3VRY4 11111

E3VS02 -------------

E3VRY5 11111 -------------

E3VRZ0 11111

E3VRY8 11111 -------------

E3VRZ1 11111 -------------

C3VLD3 11111

E3VRY9 11111

**ALIGN ****.*******************************************************************.**************************************************.******************* *****

E3VRZ5 -------------- 11111 11111 11111

E3VRZ6 -------------- 11111 11111 11111

E3VRZ4 -------------- 11111 11111 11111

E3VRY6 -------------- 11111 11111 11111

E3VRZ2 -------------- 11111 11111 11111

E3VRY7 -------------- 11111 11111 11111

E3VRZ7 -------------- 11111

E3VRZ8 -------------- 11111

E3VRZ9 -------------- 11111

E3VRZ3 -------------- 11111 11111 11111

E3VS03 -------------- 11111

E3VS04 -------------- 11111

E3VS00 -------------- 11111

E3VS01 -------------- 11111

E3VRY4 11111 11111 11111

E3VS02 -------------- 11111

E3VRY5 -------------- 11111 11111 11111

E3VRZ0 11111 11111 11111

E3VRY8 -------------- 11111 11111 11111

E3VRZ1 -------------- 11111 11111 11111

C3VLD3 11111 11111 11111

E3VRY9 11111 11111 11111

**ALIGN **********************************************.**********************.******** *************************************************.*******************

E3VRZ5 111 -----------------------------------------------------

E3VRZ6 111 -----------------------------------------------------

E3VRZ4 111 -----------------------------------------------------

E3VRY6 -----------------------------------------------------

E3VRZ2 -----------------------------------------------------

E3VRY7 -----------------------------------------------------

E3VRZ7 -----------------------------------------------------

E3VRZ8 -----------------------------------------------------

E3VRZ9 -----------------------------------------------------

E3VRZ3 -----------------------------------------------------

E3VS03 -----------------------------------------------------

E3VS04 -----------------------------------------------------

E3VS00 ------------------------------------------------------

E3VS01 ------------------------------------------------------

E3VRY4 ------------------------------------------------------

E3VS02 ------------------------------------------------------

E3VRY5 111111111

E3VRZ0 111111111

E3VRY8 111111111

E3VRZ1 - 111111111

C3VLD3 - 111111111

E3VRY9 - 111111111

**ALIGN *******************.*******************************************************.***.**

# Aligned ligand binding sites (LIG) annotated using ELMs for BK channel variants

E3VRZ5 1111111111111 11111111 11111

E3VRZ6 1111111111111 11111111 11111

E3VRZ4 1111111111111 11111111 11111

E3VRY6 1111111111111 11111111 11111

E3VRZ2 1111111111111 11111111 11111

E3VRY7 1111111111111 11111111 11111

E3VRZ7

E3VRZ8

E3VRZ9

E3VRZ3 1111111111111 11111111 11111

E3VS03

E3VS04

E3VS00

E3VS01

E3VRY4 1111111111111 11111111 11111

E3VS02

E3VRY5 1111111111111 11111111 ------------------------------------------------------ 11111

E3VRZ0 1111111111111 11111111 11111

E3VRY8 1111111111111 11111111 11111

E3VRZ1 1111111111111 11111111 11111

C3VLD3 1111111111111 11111111 11111

E3VRY9 1111111111111 11111111 11111

**ALIGN *********.*****************************.********************* ***********************************************

E3VRZ5 1111111 1111 111111111111111111111111

E3VRZ6 1111111 1111 111111111111111111111111

E3VRZ4 1111111 1111 111111111111111111111111

E3VRY6 1111111 1111 111111111111111111111111

E3VRZ2 1111111 1111 111111111111111111111111

E3VRY7 1111111 1111 111111111111111111111111

E3VRZ7

E3VRZ8

E3VRZ9

E3VRZ3 1111111 1111 111111111111111111111111

E3VS03

E3VS04

E3VS00

E3VS01

E3VRY4 1111111 1111 111111111111111111111111

E3VS02

E3VRY5 1111111 1111 111111111111111111111111

E3VRZ0 1111111 1111 111111111111111111111111

E3VRY8 1111111 1111 111111111111111111111111

E3VRZ1 1111111 1111 111111111111111111111111

C3VLD3 1111111 1111 111111111111111111111111

E3VRY9 1111111 1111 111111111111111111111111

**ALIGN ***********************************.*******************************************.**************************************** ***********************.*********** *****

E3VRZ5

E3VRZ6

E3VRZ4

E3VRY6

E3VRZ2

E3VRY7

E3VRZ7

E3VRZ8

E3VRZ9

E3VRZ3

E3VS03

E3VS04

E3VS00

E3VS01

E3VRY4 1111111111111111111 111111111 1111111 1111 1111111111111111

E3VS02

E3VRY5

E3VRZ0 1111111111111111111 111111111 1111111 1111 1111111111111111

E3VRY8

E3VRZ1

C3VLD3

E3VRY9

**ALIGN ** ** **.*.**... .**************************************.***********************.************.******************************************************************

E3VRZ5 1111111 111111111111111111 1111111 111111111 1----111 1111 1111111 -- -

E3VRZ6 1111111 111111111111111111 1111111 111111111 1----111 1111 1111111 -- 1111

E3VRZ4 1111111 111111111111111111 1111111 111111111 1----111 1111 1111111 -- -

E3VRY6 1111111 111111111111111111 1111111 111111111 1----111 1111 1111111 --

E3VRZ2 1111111 111111111111111111 1111111 111111111 11111111111 1111111111 11111

E3VRY7 1111111 111111111111111111 1111111 111111111 1----111 1111 1111111 --

E3VRZ7 -- --

E3VRZ8 --

E3VRZ9 ---- -- --

E3VRZ3 1111111 111111111111111111 1111111 111111111 1----111 1111 1111111 -- --

E3VS03 ---- --

E3VS04 ---- -- -

E3VS00 ---- --

E3VS01 ---- --

E3VRY4 1111111 111111111111111111 1111111 111111111 1----111 1111 1111111 -- 11111

E3VS02 ---- -- --

E3VRY5 1111111 111111111111111111 1111111 111111111 1----111 1111 1111111 -- 11111

E3VRZ0 1111111 111111111111111111 1111111 111111111 1----111 1111 1111111 -- 11111

E3VRY8 1111111 111111111111111111 1111111 111111111 1----111 1111 1111111 -- 11111

E3VRZ1 1111111 111111111111111111 1111111 111111111 1----111 1111 1111111 -- 11111

C3VLD3 1111111 111111111111111111 1111111 111111111 1----111 1111 1111111 -- 11111

E3VRY9 1111111 111111111111111111 1111111 111111111 1----111 1111 1111111 -- --

**ALIGN ***************..*****************.**** ******************** ************************************* ******************************** ... **..* .* ***

E3VRZ5 ------------------------------------------------------------ 111111 11111111111111111111 11111111 1111111

E3VRZ6 --------------------------------1 111 1111111111111 111111 11111111111111111111 11111111 1111111

E3VRZ4 ------------------------------------------------------------ 111111 11111111111111111111 11111111 1111111

E3VRY6 ------------------------------------------------------------- 111111 11111111111111111111 11111111 1111111

E3VRZ2 -------------------------------- 111111111 --- 11111111111111111111 11111111 1111111

E3VRY7 ------------------------------------------------------------- 111111 11111111111111111111 11111111111111 1111111

E3VRZ7 -

E3VRZ8

E3VRZ9 -

E3VRZ3 ----------------------------------------------------------- 111111 11111111111111111111 11111111 1111111

E3VS03 --------------------------------

E3VS04 ------------------------------------------------------------

E3VS00 -------------------------------------------------------------

E3VS01 -------------------------------------------------------------

E3VRY4 ----------------------------------------------------------1111 111111 11111111111111111111 11111111 1111111

E3VS02 -----------------------------------------------------------

E3VRY5 ----------------------------------------------------------1111 111111 11111111111111111111 11111111 1111111

E3VRZ0 ----------------------------------------------------------1111 111111 11111111111111111111 11111111 1111111

E3VRY8 ----------------------------------------------------------1111 111111 11111111111111111111 11111111 1111111

E3VRZ1 ----------------------------------------------------------1111 111111 11111111111111111111 11111111 1111111

C3VLD3 ----------------------------------------------------------1111 111111 11111111111111111111 11111111 1111111

E3VRY9 ----------------------------------------------------------- 111111 11111111111111111111 11111111 1111111

**ALIGN ** .******************************************************************* *************.*********

E3VRZ5 11111111111 11111111 1111111111 11111111111111111111 1111 111111111111 11111111 1111111111 111111111111-------------

E3VRZ6 11111111111 11111111 1111111111 11111111111111111111 1111 111111111111 11111111 1111111111 111111111111-------------

E3VRZ4 11111111111 11111111 1111111111 11111111111111111111 1111 111111111111 11111111 1111111111 111111111111-------------

E3VRY6 11111111111 11111111 1111111111 11111111111111111111 1111 111111111111 11111111 1111111111 111111111111-------------

E3VRZ2 11111111111 11111111 1111111111 11111111111111111111 1111 111111111111 11111111 1111111111 111111111111-------------

E3VRY7 11111111111 11111111 1111111111 11111111111111111111 1111 111111111111 11111111 1111111111 111111111111-------------

E3VRZ7 -------------

E3VRZ8 -------------

E3VRZ9 -------------

E3VRZ3 11111111111 11111111 1111111111 11111111111111111111 1111 111111111111 11111111 1111111111 111111111111-------------

E3VS03 -------------

E3VS04 -------------

E3VS00 -------------

E3VS01 -------------

E3VRY4 11111111111 11111111 1111111111 11111111111111111111 1111 111111111111 11111111 1111111111 1111111111111 1111111111

E3VS02 -------------

E3VRY5 11111111111 11111111 1111111111 11111111111111111111 1111 111111111111 11111111 1111111111 111111111111-------------

E3VRZ0 11111111111 11111111 1111111111 11111111111111111111 1111 111111111111 11111111 1111111111 1111111111111 1111111111

E3VRY8 11111111111 11111111 1111111111 11111111111111111111 1111 111111111111 11111111 1111111111 111111111111-------------

E3VRZ1 11111111111 11111111 1111111111 11111111111111111111 1111 111111111111 11111111 1111111111 111111111111-------------

C3VLD3 11111111111 11111111 1111111111 11111111111111111111 1111 111111111111 11111111 1111111111 1111111111111 1111111111

E3VRY9 11111111111 11111111 1111111111 11111111111111111111 1111 111111111111 11111111 1111111111 1111111111111 1111111111

**ALIGN ****.*******************************************************************.**************************************************.******************* *****

E3VRZ5 --------------1111 1111 111111 1111 1111 11111111 111111 11111111111 11111111111 111111

E3VRZ6 --------------1111 1111 111111 1111 1111 1111111111111111111111111 11111111111 11111111111 111111

E3VRZ4 --------------1111 1111 111111 1111 1111 1111111111111111111111111 11111111111 11111111111 111111

E3VRY6 --------------1111 1111 111111 1111 1111 1111111111111111111111111 11111111111 11111111111 111111

E3VRZ2 --------------1111 1111 111111 1111 1111 1111111111111111111111111 11111111111 11111111111 111111

E3VRY7 --------------1111 1111 111111 1111 1111 1111111111111111111111111 11111111111 11111111111 111111

E3VRZ7 --------------

E3VRZ8 --------------

E3VRZ9 --------------

E3VRZ3 --------------1111 1111 111111 1111 1111 1111111111111111111111111 11111111111 11111111111 111111

E3VS03 --------------

E3VS04 --------------

E3VS00 --------------

E3VS01 --------------

E3VRY4 1111111111111 1111 111111 1111 1111 1111111111111111111111111 11111111111 11111111111 111111

E3VS02 --------------

E3VRY5 --------------1111 1111 111111 1111 1111 1111111111111111111111111 11111111111 11111111111 111111

E3VRZ0 1111111111111 1111 111111 1111 1111 1111111111111111111111111 11111111111 11111111111 111111

E3VRY8 --------------1111 1111 111111 1111 1111 1111111111111111111111111 11111111111 11111111111 111111

E3VRZ1 --------------1111 1111 111111 1111 1111 1111111111111111111111111 11111111111 11111111111 111111

C3VLD3 1111111111111 1111 111111 1111 1111 1111111111111111111111111 11111111111 11111111111 111111

E3VRY9 1111111111111 1111 111111 1111 1111 1111111111111111111111111 11111111111 11111111111 111111

**ALIGN **********************************************.**********************.******** *************************************************.*******************

E3VRZ5 1111111111111111111 11111 11111 1111111 1111111-----------------------------------------------------

E3VRZ6 1111111111111111111 11111 11111 1111111 1111111-----------------------------------------------------

E3VRZ4 1111111111111111111 11111 11111 1111111 1111111-----------------------------------------------------

E3VRY6 1111111111111111111 11111 11111 1111111 1111111-----------------------------------------------------

E3VRZ2 11111111111111 11111 11111 1111111 1111111-----------------------------------------------------

E3VRY7 1111111111111111111 11111 11111 1111111 1111111-----------------------------------------------------

E3VRZ7 -----------------------------------------------------

E3VRZ8 -----------------------------------------------------

E3VRZ9 -----------------------------------------------------

E3VRZ3 1111111111111111111 11111 11111 1111111 -----------------------------------------------------

E3VS03 -----------------------------------------------------

E3VS04 -----------------------------------------------------

E3VS00 ------------------------------------------------------

E3VS01 ------------------------------------------------------

E3VRY4 1111111111111111111 11111 11111 1111111 ------------------------------------------------------

E3VS02 ------------------------------------------------------

E3VRY5 1111111111111111111 11111 11111 1111111 111111111111111111

E3VRZ0 1111111111111111111 11111 11111 1111111 111111111111111111

E3VRY8 1111111111111111111 11111 11111 1111111 111111111111111111

E3VRZ1 1111111111111111111 11111 11111 1111111 - 111111111111111111

C3VLD3 1111111111111111111 11111 11111 1111111 - 111111111111111111

E3VRY9 1111111111111111111 11111 11111 1111111 - 111111111111111111

**ALIGN *******************.*******************************************************.***.**

# Aligned posttranslational modification sites (MOD) annotated using ELMs for BK channel variants

E3VRZ5 1111111111111 111111 11111111

E3VRZ6 1111111111111 111111 11111111

E3VRZ4 1111111111111 111111 11111111

E3VRY6 1111111111111 111111 1111111

E3VRZ2 1111111111111 111111 1111111

E3VRY7 1111111111111 111111 1111111

E3VRZ7 111111

E3VRZ8 111111

E3VRZ9 111111

E3VRZ3 1111111111111 111111 1111111

E3VS03 111111

E3VS04 111111

E3VS00 111111

E3VS01 111111

E3VRY4 1111111111111 111111 1111111

E3VS02 111111

E3VRY5 ------------------------------------------------------

E3VRZ0 1111111111111 111111 1111111

E3VRY8 1111111111111 111111 1111111

E3VRZ1 1111111111111 111111 1111111

C3VLD3 1111111111111 111111 1111111

E3VRY9 1111111111111 111111 1111111

**ALIGN *********.*****************************.********************* ***********************************************

E3VRZ5 111111 111111

E3VRZ6 111111 111111

E3VRZ4 111111 111111

E3VRY6 111111 111111

E3VRZ2 111111 111111

E3VRY7 111111 111111

E3VRZ7 111111

E3VRZ8 111111

E3VRZ9 111111

E3VRZ3 111111 111111

E3VS03 111111

E3VS04 111111

E3VS00 111111

E3VS01 111111

E3VRY4 111111 111111

E3VS02 111111

E3VRY5 111111 111111

E3VRZ0 111111 111111

E3VRY8 111111 111111

E3VRZ1 111111 111111

C3VLD3 111111 111111

E3VRY9 111111 111111

**ALIGN ***********************************.*******************************************.**************************************** ***********************.*********** *****

E3VRZ5 11111111

E3VRZ6 11111111

E3VRZ4 11111111

E3VRY6 111111

E3VRZ2 1111111

E3VRY7 1111111

E3VRZ7

E3VRZ8

E3VRZ9

E3VRZ3 1111111

E3VS03

E3VS04

E3VS00

E3VS01

E3VRY4 1111111 1111111 1111111 1111 1111111 1111111

E3VS02

E3VRY5 1111111

E3VRZ0 1111111 1111111 1111111111111 1111 1111111 1111111

E3VRY8 1111111

E3VRZ1 1111111

C3VLD3 1111111

E3VRY9 1111111

**ALIGN ** ** **.*.**... .**************************************.***********************.************.******************************************************************

E3VRZ5 111111111111111 11111111111 1111111 11111111----11 1111111 -- -

E3VRZ6 111111111111111 11111111111 1111111 11111111----11 1111111 -- 11

E3VRZ4 111111111111111 11111111111 1111111 11111111----11 1111111 -- -

E3VRY6 111111111111111 11111111 1111111 11111----11 1111111 --

E3VRZ2 111111111111111 11111111 1111111 1111111 1111111 1111111 11

E3VRY7 111111111111111 11111111 1111111 11111----11 1111111 --

E3VRZ7 -- --

E3VRZ8 --

E3VRZ9 ---- -- --

E3VRZ3 111111111111111 11111111 1111111 11111----11 1111111 -- --

E3VS03 ---- --

E3VS04 ---- -- -

E3VS00 ---- --

E3VS01 ---- --

E3VRY4 111111111111111 11111111 1111111 11111----11 1111111 --

E3VS02 ---- -- --

E3VRY5 111111111111111 11111111 1111111 11111----11 1111111 --

E3VRZ0 111111111111111 11111111 1111111 11111----11 1111111 --

E3VRY8 111111111111111 11111111 1111111 11111----11 1111111 --

E3VRZ1 111111111111111 11111111 1111111 11111----11 1111111 --

C3VLD3 111111111111111 11111111 1111111 11111----11 1111111 --

E3VRY9 111111111111111 11111111 1111111 11111----11 1111111 -- --

**ALIGN ***************..*****************.**** ******************** ************************************* ******************************** ... **..* .* ***

E3VRZ5 ------------------------------------------------------------ 1111111 11111111111 1111111 1111 1111

E3VRZ6 --------------------------------11 1111111111 1111111 11111111111 1111111 1111 1111

E3VRZ4 ------------------------------------------------------------ 1111111 11111111111 1111111 1111 1111

E3VRY6 ------------------------------------------------------------- 1111111 11111111111 1111111

E3VRZ2 --------------------------------11 11111111 11111111111 --- 11111111111 1111111

E3VRY7 ------------------------------------------------------------- 1111111 11111111111 11111111111111

E3VRZ7 - 111 111111

E3VRZ8 111 111111

E3VRZ9 - 111 111111

E3VRZ3 ----------------------------------------------------------- 1111111 11111111111 1111111

E3VS03 -------------------------------- 111111 111111

E3VS04 ------------------------------------------------------------ 111111

E3VS00 ------------------------------------------------------------- 111111

E3VS01 ------------------------------------------------------------- 111111

E3VRY4 ---------------------------------------------------------- 1111111 11111111111 1111111

E3VS02 ----------------------------------------------------------- 111111

E3VRY5 ---------------------------------------------------------- 1111111 11111111111 1111111

E3VRZ0 ---------------------------------------------------------- 1111111 11111111111 1111111

E3VRY8 ---------------------------------------------------------- 1111111 11111111111 1111111

E3VRZ1 ---------------------------------------------------------- 1111111 11111111111 1111111

C3VLD3 ---------------------------------------------------------- 1111111 11111111111 1111111

E3VRY9 ----------------------------------------------------------- 1111111 11111111111 1111111

**ALIGN ** .******************************************************************* *************.*********

E3VRZ5 1111111 1111 1111111 111 1111 1111111 1111111 11111111111111111111111 1111111111 -------------

E3VRZ6 1111111 1111 1111111 111 1111 1111111 1111111 11111111111111111111111 1111111111 -------------

E3VRZ4 1111111 1111 1111111 111 1111 1111111 1111111 11111111111111111111111 1111111111 -------------

E3VRY6 1111111 1111 1111111 111 1111111 1111111 11111111111111111111111 1111111111 -------------

E3VRZ2 1111111 1111 1111111 111 1111111 1111111 11111111111111111111111 1111111111 -------------

E3VRY7 1111111 1111 1111111 111 1111111 1111111 11111111111111111111111 1111111111 -------------

E3VRZ7 111 -------------

E3VRZ8 111 -------------

E3VRZ9 111 -------------

E3VRZ3 1111111 1111 1111111 111 1111111 1111111 11111111111111111111111 1111111111 -------------

E3VS03 111 -------------

E3VS04 111 -------------

E3VS00 111 -------------

E3VS01 111 -------------

E3VRY4 1111111 1111 1111111 111 1111111 1111111 11111111111111111111111 1111111111

E3VS02 111 -------------

E3VRY5 1111111 1111 1111111 111 1111111 1111111 11111111111111111111111 1111111111 -------------

E3VRZ0 1111111 1111 1111111 111 1111111 1111111 11111111111111111111111 1111111111

E3VRY8 1111111 1111 1111111 111 1111111 1111111 11111111111111111111111 1111111111 -------------

E3VRZ1 1111111 1111 1111111 111 1111111 1111111 11111111111111111111111 1111111111 -------------

C3VLD3 1111111 1111 1111111 111 1111111 1111111 11111111111111111111111 1111111111

E3VRY9 1111111 1111 1111111 111 1111111 1111111 11111111111111111111111 1111111111

ALIGN ****.*******************************************************************.**************************************************.******************* ***

E3VRZ5 -------------- 111111 1111111 111111111111111 111111111111111111 11111111 111111

E3VRZ6 -------------- 111111 1111111 111111111111111 111111111111111111 11111111 111111

E3VRZ4 -------------- 111111 1111111 111111111111111 111111111111111111 11111111 111111

E3VRY6 -------------- 111111 1111111 111111111111111 111111111111111111 11111111 111111

E3VRZ2 -------------- 111111 1111111 111111111111111 111111111111111111 11111111 111111

E3VRY7 -------------- 111111 1111111 111111111111111 111111111111111111 11111111 111111

E3VRZ7 -------------- 111111

E3VRZ8 -------------- 111111

E3VRZ9 -------------- 111111

E3VRZ3 -------------- 111111 1111111 111111111111111 111111111111111111 11111111 111111

E3VS03 -------------- 111111

E3VS04 -------------- 111111

E3VS00 -------------- 111111

E3VS01 -------------- 111111

E3VRY4 111111 1111111 111111111111111 111111111111111111 11111111 111111

E3VS02 -------------- 111111

E3VRY5 -------------- 111111 1111111 111111111111111 111111111111111111 11111111 111111

E3VRZ0 111111 1111111 111111111111111 111111111111111111 11111111 111111

E3VRY8 -------------- 111111 1111111 111111111111111 111111111111111111 11111111 111111

E3VRZ1 -------------- 111111 1111111 111111111111111 111111111111111111 11111111 111111

C3VLD3 111111 1111111 111111111111111 111111111111111111 11111111 111111

E3VRY9 111111 1111111 111111111111111 111111111111111111 11111111 111111

**ALIGN **********************************************.**********************.******** *************************************************.*******************

E3VRZ5 111111 111111111111111111111111111111 1111111 -----------------------------------------------------

E3VRZ6 111111 111111111111111111111111111111 1111111 -----------------------------------------------------

E3VRZ4 111111 111111111111111111111111111111 1111111 -----------------------------------------------------

E3VRY6 111111 111111111111111111111111111111 1111111 -----------------------------------------------------

E3VRZ2 111111 111111111111111111111111111111 1111111 -----------------------------------------------------

E3VRY7 111111 111111111111111111111111111111 1111111 -----------------------------------------------------

E3VRZ7 -----------------------------------------------------

E3VRZ8 -----------------------------------------------------

E3VRZ9 -----------------------------------------------------

E3VRZ3 111111 111111111111111111111111111111 111111111111 -----------------------------------------------------

E3VS03 -----------------------------------------------------

E3VS04 -----------------------------------------------------

E3VS00 ------------------------------------------------------

E3VS01 ------------------------------------------------------

E3VRY4 111111 111111111111111111111111111111 1111111 ------------------------------------------------------

E3VS02 ------------------------------------------------------

E3VRY5 111111 111111111111111111111111111111 11111111111111111 1111111

E3VRZ0 111111 111111111111111111111111111111 11111111111111111 1111111

E3VRY8 111111 111111111111111111111111111111 11111111111111111 1111111

E3VRZ1 111111 111111111111111111111111111111 11111111111111111 - 1111111

C3VLD3 111111 111111111111111111111111111111 11111111111111111 - 1111111

E3VRY9 111111 111111111111111111111111111111 11111111111111111 - 1111111

**ALIGN *******************.*******************************************************.***.**

# Aligned subcellular target sites (TRG) annotated using ELMs for BK channel variants

E3VRZ5

E3VRZ6

E3VRZ4

E3VRY6

E3VRZ2

E3VRY7

E3VRZ7

E3VRZ8

E3VRZ9

E3VRZ3

E3VS03

E3VS04

E3VS00

E3VS01

E3VRY4

E3VS02

E3VRY5 ------------------------------------------------------

E3VRZ0

E3VRY8

E3VRZ1

C3VLD3

E3VRY9

**ALIGN *********.*****************************.********************* ***********************************************

E3VRZ5 111111

E3VRZ6 111111

E3VRZ4 111111

E3VRY6 111111

E3VRZ2 111111

E3VRY7 111111

E3VRZ7

E3VRZ8

E3VRZ9

E3VRZ3 111111

E3VS03

E3VS04

E3VS00

E3VS01

E3VRY4 111111

E3VS02

E3VRY5 111111

E3VRZ0 111111

E3VRY8 111111

E3VRZ1 111111

C3VLD3 111111

E3VRY9 111111

**ALIGN ***********************************.*******************************************.**************************************** ***********************.*********** *****

E3VRZ5 1111

E3VRZ6 1111

E3VRZ4 1111

E3VRY6 11111

E3VRZ2 1111

E3VRY7 1111

E3VRZ7

E3VRZ8

E3VRZ9

E3VRZ3 1111

E3VS03

E3VS04

E3VS00

E3VS01

E3VRY4 1111 11111 11111

E3VS02

E3VRY5 1111

E3VRZ0 1111 11111 11111

E3VRY8 1111

E3VRZ1 1111

C3VLD3 1111

E3VRY9 1111

**ALIGN ** ** **.*.**... .**************************************.***********************.************.******************************************************************

E3VRZ5 11111 111111111111111 111----111 1111111111111111111111--111111-

E3VRZ6 11111 111111111111111 111----111 1111111111111111111111-- 111

E3VRZ4 11111 111111111111111 111----111 1111111111111111111111--111111-

E3VRY6 11111 111111111111111 111----111 1111111111111111111111--1111111

E3VRZ2 11111 111111111111111 11111111

E3VRY7 11111 111111111111111 111----111 1111111111111111111111--1111111

E3VRZ7 -- --

E3VRZ8 --

E3VRZ9 ---- -- --

E3VRZ3 11111 111111111111111 111----111 1111111111111111111111--11111--

E3VS03 ---- --

E3VS04 ---- -- -

E3VS00 ---- --

E3VS01 ---- --

E3VRY4 11111 111111111111111 111----111 1111111111111111111111-- 1111

E3VS02 ---- -- --

E3VRY5 11111 111111111111111 111----111 1111111111111111111111-- 1111

E3VRZ0 11111 111111111111111 111----111 1111111111111111111111-- 1111

E3VRY8 11111 111111111111111 111----111 1111111111111111111111-- 1111

E3VRZ1 11111 111111111111111 111----111 1111111111111111111111-- 1111

C3VLD3 11111 111111111111111 111----111 1111111111111111111111-- 1111

E3VRY9 11111 111111111111111 111----111 1111111111111111111111--11111--

**ALIGN ***************..*****************.**** ******************** ************************************* ******************************** ... **..* .* ***

E3VRZ5 ------------------------------------------------------------1111111111111111 111111

E3VRZ6 -------------------------------- 1111 1111111 111111

E3VRZ4 ------------------------------------------------------------1111111111111111 111111

E3VRY6 -------------------------------------------------------------111111111111111 111111

E3VRZ2 -------------------------------- 1111 --- 111111

E3VRY7 -------------------------------------------------------------111111111111111 111111

E3VRZ7 -

E3VRZ8

E3VRZ9 -

E3VRZ3 -----------------------------------------------------------11111111111111111 111111

E3VS03 --------------------------------

E3VS04 ------------------------------------------------------------

E3VS00 -------------------------------------------------------------

E3VS01 -------------------------------------------------------------

E3VRY4 ----------------------------------------------------------111111111111111111 111111

E3VS02 -----------------------------------------------------------

E3VRY5 ----------------------------------------------------------111111111111111111 111111

E3VRZ0 ----------------------------------------------------------111111111111111111 111111

E3VRY8 ----------------------------------------------------------111111111111111111 111111

E3VRZ1 ----------------------------------------------------------111111111111111111 111111

C3VLD3 ----------------------------------------------------------111111111111111111 111111

E3VRY9 -----------------------------------------------------------11111111111111111 111111

**ALIGN ** .******************************************************************* *************.*********

E3VRZ5 1111 -------------

E3VRZ6 1111 -------------

E3VRZ4 1111 -------------

E3VRY6 1111 -------------

E3VRZ2 1111 -------------

E3VRY7 1111 -------------

E3VRZ7 -------------

E3VRZ8 -------------

E3VRZ9 -------------

E3VRZ3 1111 -------------

E3VS03 -------------

E3VS04 -------------

E3VS00 -------------

E3VS01 -------------

E3VRY4 1111

E3VS02 -------------

E3VRY5 1111 -------------

E3VRZ0 1111

E3VRY8 1111 -------------

E3VRZ1 1111 -------------

C3VLD3 1111

E3VRY9 1111

**ALIGN ****.*******************************************************************.**************************************************.******************* *****

E3VRZ5 -------------- 111111 1111 1111 1111 1111

E3VRZ6 -------------- 111111 1111 1111 1111 1111

E3VRZ4 -------------- 111111 1111 1111 1111 1111

E3VRY6 -------------- 111111 1111 1111 1111 1111

E3VRZ2 -------------- 111111 1111 1111 1111 1111

E3VRY7 -------------- 111111 1111 1111 1111 1111

E3VRZ7 --------------

E3VRZ8 --------------

E3VRZ9 --------------

E3VRZ3 -------------- 111111 1111 1111 1111 1111

E3VS03 --------------

E3VS04 --------------

E3VS00 --------------

E3VS01 --------------

E3VRY4 111111 1111 1111 1111 1111

E3VS02 --------------

E3VRY5 -------------- 111111 1111 1111 1111 1111

E3VRZ0 111111 1111 1111 1111 1111

E3VRY8 -------------- 111111 1111 1111 1111 1111

E3VRZ1 -------------- 111111 1111 1111 1111 1111

C3VLD3 111111 1111 1111 1111 1111

E3VRY9 111111 1111 1111 1111 1111

ALIGN **********************************************.**********************.******** *************************************************.*****************

E3VRZ5 -----------------------------------------------------

E3VRZ6 -----------------------------------------------------

E3VRZ4 -----------------------------------------------------

E3VRY6 -----------------------------------------------------

E3VRZ2 -----------------------------------------------------

E3VRY7 -----------------------------------------------------

E3VRZ7 -----------------------------------------------------

E3VRZ8 -----------------------------------------------------

E3VRZ9 -----------------------------------------------------

E3VRZ3 -----------------------------------------------------

E3VS03 -----------------------------------------------------

E3VS04 -----------------------------------------------------

E3VS00 ------------------------------------------------------

E3VS01 ------------------------------------------------------

E3VRY4 ------------------------------------------------------

E3VS02 ------------------------------------------------------

E3VRY5

E3VRZ0

E3VRY8

E3VRZ1 -

C3VLD3 -

E3VRY9 -

**ALIGN *******************.*******************************************************.***.**
