# Supplementary material for: Intrinsic Disorder in the BK Channel and Its Interactome
Source: PLoS One. 2014 Apr 11;9(4):e94331. doi: 10.1371/journal.pone.0094331 (PMC3984161; doi:10.1371/journal.pone.0094331)
Supplement: Table S1 — Aligned ELM annotations for BK channel variants. Grouping of 59 ELM classes into 4 types including cleavage sites (CLV), ligand binding sites (LIG), sites of posttranslational modification (MOD) and subcellular targeting sites (TRG). (PDF) [file pone.0094331.s001.pdf]

Aligned ELM annotations for BK channel variants


```
CLV_PCSK_SKI1_1:22
E3VRZ5    11111
E3VRZ6    11111
E3VRZ4    11111
E3VRY6    11111
E3VRZ2    11111
E3VRY7    11111
E3VRZ7    11111
E3VRZ8    11111
E3VRZ9    11111
E3VRZ3    11111
E3VS03    11111
E3VS04    11111
E3VS00    11111
E3VS01    11111
E3VRY4    11111
E3VS02    11111
E3VRY5    11111
E3VRZ0    11111
E3VRY8    11111
E3VRZ1    11111
C3VLD3    11111
E3VRY9    11111
ALIGN
MOD_N-GLC_1:22
E3VRZ5    111111    111111
E3VRZ6    111111    111111
E3VRZ4    111111    111111
E3VRY6    111111    111111
E3VRZ2    111111    111111
E3VRY7    111111    111111
E3VRZ7    111111    111111
E3VRZ8    111111    111111
E3VRZ9    111111    111111
E3VRZ3    111111    111111
E3VS03    111111    111111
E3VS04    111111    111111
E3VS00    111111    111111
E3VS01    111111    111111
E3VRY4    111111    111111
E3VS02    111111    111111
E3VRY5    111111
E3VRZ0    111111    111111
E3VRY8    111111    111111
E3VRZ1    111111    111111
C3VLD3    111111    111111
E3VRY9    111111    111111
ALIGN
MOD_N-GLC_2:22
E3VRZ5
E3VRZ6
E3VRZ4
E3VRY6
E3VRZ2
E3VRY7
E3VRZ7
E3VRZ8
E3VRZ9
E3VRZ3
E3VS03
E3VS04
E3VS00
E3VS01
E3VRY4
E3VS02
E3VRY5
E3VRZ0
E3VRY8
E3VRZ1
C3VLD3
E3VRY9
ALIGN
CLV_C14_Caspase3-7:14
E3VRZ5    11111
E3VRZ6    11111
E3VRZ4    11111
E3VRY6    11111
E3VRZ2    11111
E3VRY7    11111
E3VRZ7
E3VRZ8
E3VRZ9
E3VRZ3    11111
E3VS03
E3VS04
E3VS00
E3VS01
E3VRY4    11111
E3VS02
E3VRY5
E3VRZ0    11111
E3VRY8    11111
E3VRZ1    11111
C3VLD3    11111
E3VRY9    11111
ALIGN
```
