# Supplementary material for: Intrinsic Disorder in the BK Channel and Its Interactome
Source: PLoS One. 2014 Apr 11;9(4):e94331. doi: 10.1371/journal.pone.0094331 (PMC3984161; doi:10.1371/journal.pone.0094331)
Supplement: Table S2 — Aligned ClustalW annotations for overall characterization of BK channel variants. Sequence profiles of BK channel variants showing ID, MoRF regions, globular domains, and the four types of ELMs. (PDF) [file pone.0094331.s002.pdf]

Annotations of the aligned set of BK channel variants

ALIGN: results of ClustalW alignment
  * identical residues
  . mutation/substitution
  blank insertion/deletion
conSQ: consensus sequence (based on the most frequent residue type at each position in the ClustalW alignment)
MUTAT: number of different residue types for positions with mutations/substitutions
INSER: - indicates insertions due to alternative splicing
DISOR: predicted disorder (based on majority vote over the considered variants)
  1 disordered residues
  0 structured residues
MoRF: predicted MoRF residues (based on majority vote over the considered variants)
  1 MoRF residues
  0 non-MoRF residues
GlobD: predicted globular domains (based on majority vote over the considered variants)
  1 residue in a globular domain
  0 residue not in a globular domain
CLV_: cleavage sites annotated with ELMs
  1 residue in a cleavage site
  0 residue that was not found to be in a cleavage site based on ELMs
LIG_: ligand binding sites annotated with ELMs
  1 residue in a ligand binding site
  0 residue that was not found to be in a ligand binding site based on ELMs
MOD_: posttranslational modification sites annotated with ELMs
  1 posttranslational modification site
  0 residue that was not found to be in a posttranslational modification site based on ELMs
TRG_: subcellular targeting sites annotated with ELMs
  1 residue in a subcellular targeting site
  0 residue that was not found to be in a subcellular targeting site based on ELMs

Results across all BK channel variants

```
ALIGN [illegible]
conSQ [illegible]
MUTAT [illegible]
INSER [illegible]
DISOR [illegible]
MoRF  [illegible]
GlobD [illegible]
CLV_  [illegible]
LIG_  [illegible]
MOD_  [illegible]
TRG_  [illegible]
```

Results across DEC variants

```
ALIGN [illegible]
conSQ [illegible]
MUTAT [illegible]
INSER [illegible]
DISOR [illegible]
MoRF  [illegible]
GlobD [illegible]
CLV_  [illegible]
LIG_  [illegible]
MOD_  [illegible]
TRG_  [illegible]
```

Results across ERL variants

```
ALIGN [illegible]
conSQ [illegible]
MUTAT [illegible]
INSER [illegible]
DISOR [illegible]
MoRF  [illegible]
GlobD [illegible]
CLV_  [illegible]
LIG_  [illegible]
MOD_  [illegible]
TRG_  [illegible]
```

Results across VYR variants

```
ALIGN [illegible]
conSQ [illegible]
MUTAT [illegible]
INSER [illegible]
DISOR [illegible]
MoRF  [illegible]
GlobD [illegible]
CLV_  [illegible]
LIG_  [illegible]
MOD_  [illegible]
TRG_  [illegible]
```
